# Identification of Novel Rotihibin Analogues in *Streptomyces scabies*, Including Discovery of Its Biosynthetic Gene Cluster

**DOI:** 10.1128/spectrum.00571-21

**Published:** 2021-08-04

**Authors:** Sören Planckaert, Benoit Deflandre, Anne-Mare de Vries, Maarten Ameye, José C. Martins, Kris Audenaert, Sébastien Rigali, Bart Devreese

**Affiliations:** a Laboratory for Microbiology, Department of Biochemistry and Microbiology, Ghent University, Ghent, Belgium; b InBioS-Centre for Protein Engineering, Institut de Chimie B6a, University of Liège, Liège, Belgium; c NMR and Structure Analysis Group, Ghent University, Ghent, Belgium; d Laboratory of Applied Mycology and Phenomics, Department of Plants and Crops, Ghent University, Ghent, Belgium; University of Minnesota

**Keywords:** *Streptomyces*, TORK, common scab, lipopeptide, nonribosomal peptide, proteomics

## Abstract

Streptomyces scabies is a phytopathogen associated with common scab disease. This is mainly attributed to its ability to produce the phytotoxin thaxtomin A, the biosynthesis of which is triggered by cellobiose. During a survey of other metabolites released in the presence of cellobiose, we discovered additional compounds in the thaxtomin-containing extract from *Streptomyces scabies*. Structural analysis by mass spectrometry (MS) and nuclear magnetic resonance (NMR) revealed that these compounds are amino acid sequence variants of the TOR (target of rapamycin) kinase (TORK) pathway-inhibitory lipopeptide rotihibin A, and the main compounds were named rotihibins C and D. In contrast to thaxtomin, the production of rotihibins C and D was also elicited in the presence of glucose, indicating different regulation of their biosynthesis. Through a combination of shotgun and targeted proteomics, the putative rotihibin biosynthetic gene cluster *rth* was identified in the publicly available genome of S. scabies 87-22. This cluster spans 33 kbp and encodes 2 different nonribosomal peptide synthetases (NRPSs) and 12 additional enzymes. Homologous *rth* biosynthetic gene clusters were found in other publicly available and complete actinomycete genomes. Rotihibins C and D display herbicidal activity against Lemna minor and Arabidopsis thaliana at low concentrations, shown by monitoring the effects on growth and the maximal photochemistry efficiency of photosystem II.

**IMPORTANCE** Rotihibins A and B are plant growth inhibitors acting on the TORK pathway. We report the isolation and characterization of new sequence analogues of rotihibin from *Streptomyces scabies*, a major cause of common scab in potato and other tuber and root vegetables. By combining proteomics data with genomic analysis, we found a cryptic biosynthetic gene cluster coding for enzyme machinery capable of rotihibin production. This work may lead to the biotechnological production of variants of this lipopeptide to investigate the exact mechanism by which it can target the plant TORK pathway in Arabidopsis thaliana. In addition, bioinformatics revealed the existence of other variants in plant-associated *Streptomyces* strains, both pathogenic and nonpathogenic species, raising new questions about the actual function of this lipopeptide. The discovery of a module in the nonribosomal peptide synthetase (NRPS) that incorporates the unusual citrulline residue may improve the prediction of peptides encoded by cryptic NRPS gene clusters.

## INTRODUCTION

Streptomyces scabies (syn. *S. scabiei*) is a plant-pathogenic bacterium causing common scab disease, resulting in substantial damage to potatoes and other root and tuber crops, including carrot, radish, beet, parsnip, and turnip. Common scab disease is widely distributed and seriously diminishes the market value of the crops (1). The disease is characterized by deep-pitted and corky lesions on the root or tuber surface (2). Other *Streptomyces* species, S. reticuliscabiei, S. cheloniumii, and S. ipomoeae, are responsible for netted scab, russet scab, and soil rot of sweet potato, respectively (3–5).

In the last decades, several studies focused on elucidating the molecular mechanisms of virulence of S. scabies. The production of thaxtomin A has been shown to be a key pathogenicity determinant (6). The *txtABCDEH* gene cluster is responsible for the biosynthesis of this 4-nitroindol-3-yl-containing 2,5-dioxo-piperazine. These thaxtomin biosynthetic genes are highly conserved across plant-pathogenic streptomycetes and reside on a pathogenicity island that is mobilized in some species (7). Thaxtomin A primarily targets expanding host tissue by affecting cellulose synthase complex density, expression of cell wall genes, and cell wall composition (8, 9).

The production of thaxtomin A is strictly controlled, involving several layers of regulation. Cellobiose, together with cellotriose, is recognized as the main specific elicitor of thaxtomin A biosynthesis in *S. scabies* (10). Once imported via the CebEFG-MsiK ABC transporter (11), these products of cellulose turnover directly target the pathway-specific transcriptional activator TxtR and the cellulose utilization regulator CebR, which together constitute a double-locking system on the *txtABCDE* gene cluster. Specific interaction between CebR and cellobiose triggers the release of the repressor from different binding sites within the thaxtomin biosynthetic gene cluster (BGC), including *txtR*. This results in the transcriptional activation of *txtA* and *txtB*, consequently inducing thaxtomin A production and pathogenicity (12, 13). In addition, several *bld* global regulators directing secondary metabolism or morphological differentiation are involved in the regulation of thaxtomin synthesis (14).

It is believed that plant-pathogenic streptomycetes produce other important phytotoxins involved in pathogenicity. Using proteomics, we previously demonstrated that the levels of enzymes involved in the biosynthesis of other secondary metabolites like concanamycin A and coronafacoyl phytotoxins are also dependent on cellobiose levels, a finding later confirmed by measuring altered levels of these compounds in the presence of cellobiose and/or upon *cebR* deletion (15). Natsume et al. isolated concanamycins A and B from Japanese *S. scabies* isolates (16). Recently, they demonstrated root growth-inhibitory activity, necrosis-inducing activity, and a synergistic effect with thaxtomin A (17). Coronafacoyl phytotoxins contribute to the development of root disease symptoms and cause hypertrophy of potato tuber tissue (18). The causative agent of russet scab, *S. cheloniumii*, produces FD-891, which induces necrosis of potato tuber tissue (19). All these data indicate that multiple secondary metabolites are involved in scab disease. Moreover, thaxtomin A-deficient streptomycetes that are also able to cause scab disease have been isolated. *Streptomyces* sp. strain GK18 produces borrelidin, which is reported to cause severe deep, black holes on potato tuber slices (20, 21). Similarly, fridamycin E was isolated from an S. turgidiscabies strain from a netted scab lesion in Sweden. This phytotoxin was demonstrated to reduce or even inhibit the sprouting of *in vitro* microtubers (22).

Driven by the discovery that under thaxtomin production-inducing conditions, i.e., the addition of cellobiose, multiple proteins potentially involved in secondary metabolism were increased in abundance, we further analyzed extracts from the extracellular medium of *Streptomyces scabies* RL-34 in order to obtain insight into the metabolites secreted by this organism when grown in the presence of cellobiose. We report here the discovery of two new compounds that were characterized to be variants of the lipopeptide rotihibins A and B, previously discovered by Fukuchi et al. in extracellular extracts of *Streptomyces* sp. strain 3C02, later designated an S. graminofaciens strain (23, 24). A culture filtrate containing rotihibins A and B inhibits the growth of lettuce seedlings, while purified rotihibin A causes shoot stunting in tobacco seedlings at low concentrations (25). Recently, it was demonstrated that rotihibin A acts as a TOR (target of rapamycin) kinase (TORK) pathway inhibitor (26). Therefore, we assessed the plant growth-inhibitory effect of the newly isolated compounds, designated rotihibins C and D, and found a severe effect on growth and photosystem II photochemistry efficiency in Lemna minor L. and Arabidopsis thaliana L. Heynh. Furthermore, we provide data that demonstrate that the biosynthetic machinery to produce rotihibins C and D is encoded by a gene cluster covering a 33-kb segment containing 14 open reading frames (ORFs) that is conserved in both pathogenic and nonpathogenic plant-associated *Streptomyces* species.

## RESULTS AND DISCUSSION

### Rotihibin production by *Streptomyces scabies*.

**FIG 1:**
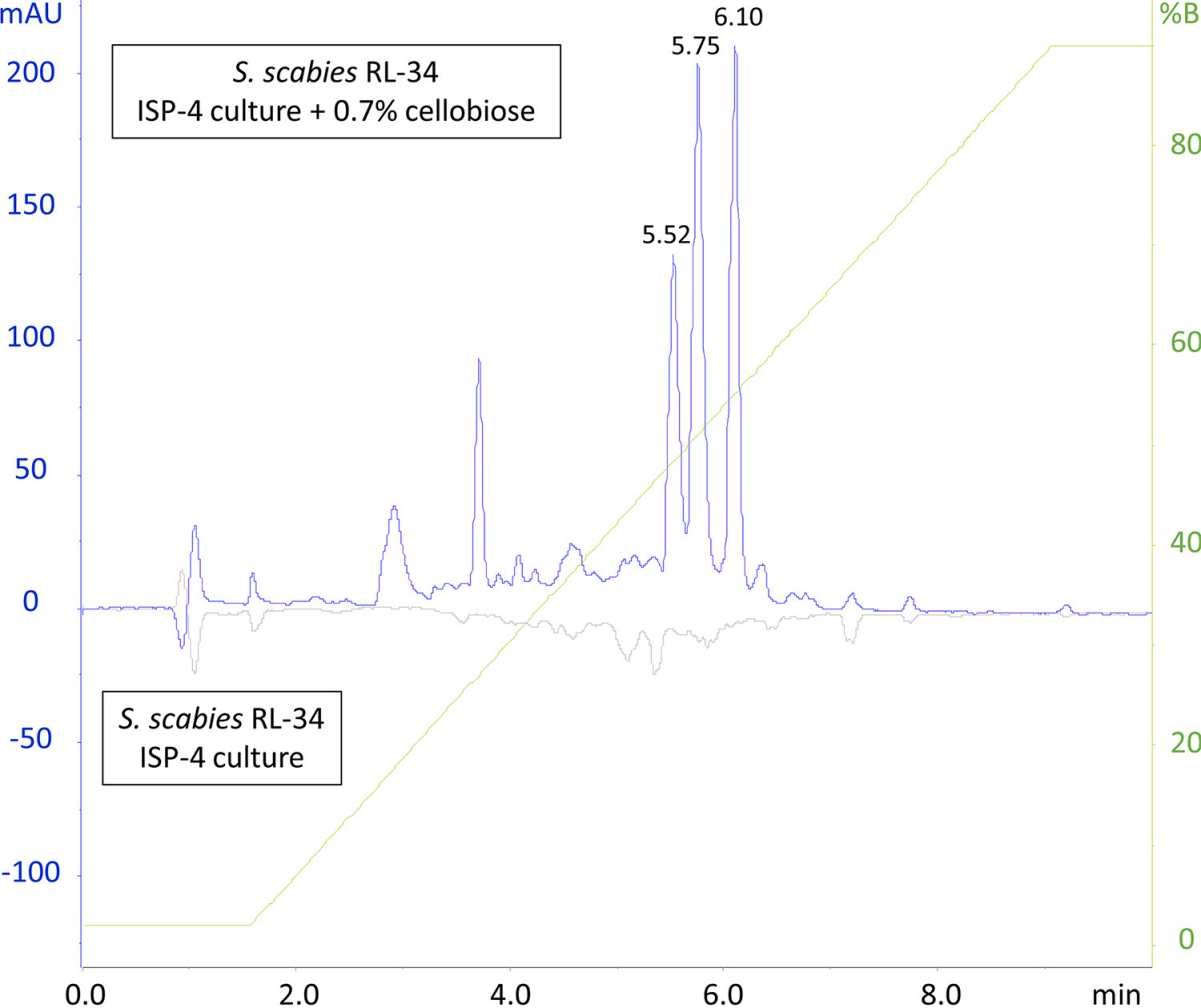
Effect of cellobiose on the secreted metabolome of *Streptomyces scabies* RL-34. HPLC runs were performed to compare *n*-butanol extracts of *Streptomyces scabies* RL-34 ISP-4 cultures grown in the presence of cellobiose (upward chromatogram) to those of cultures grown in the absence of cellobiose (downward chromatogram). mAU, milli-absorbance unit.

Cellobiose is known to induce thaxtomin A production in *Streptomyces scabies* (10). We postulated that cellobiose also elicits the production of other virulence factors important for infection of root and tuber crops. When comparing the high-performance liquid chromatography (HPLC) profiles of *n*-butanol extracts of media from cultures obtained from *S. scabies* RL-34 grown in International *Streptomyces* Project medium 4 (ISP-4) either supplemented or not with 0.7% cellobiose, new peaks appeared in the HPLC profile of the extract obtained after growth in the presence of cellobiose (Fig. 1). We focused further analysis on the peaks eluting at 5.52, 5.75, and 6.10 min, and the corresponding metabolites were characterized by high-resolution electrospray ionization mass spectrometry (HR-ESI-MS). A distinct ion peak at *m/z* 439.16 [M + H]^+^ could be identified in the spectrum obtained from the fraction eluting at 6.10 min, accompanied by peaks at *m/z* 421.15 [M − H_2_O + H]^+^, 461.14 [M + Na]^+^, and 477.11 [M + K]^+^ (data not shown). This pattern of *m/z* values can be attributed to thaxtomin A (theoretical monoisotopic mass, 438.11), confirming that our culture conditions induced the production of the main pathogenic determinant of *S. scabies*.

**FIG 2:**
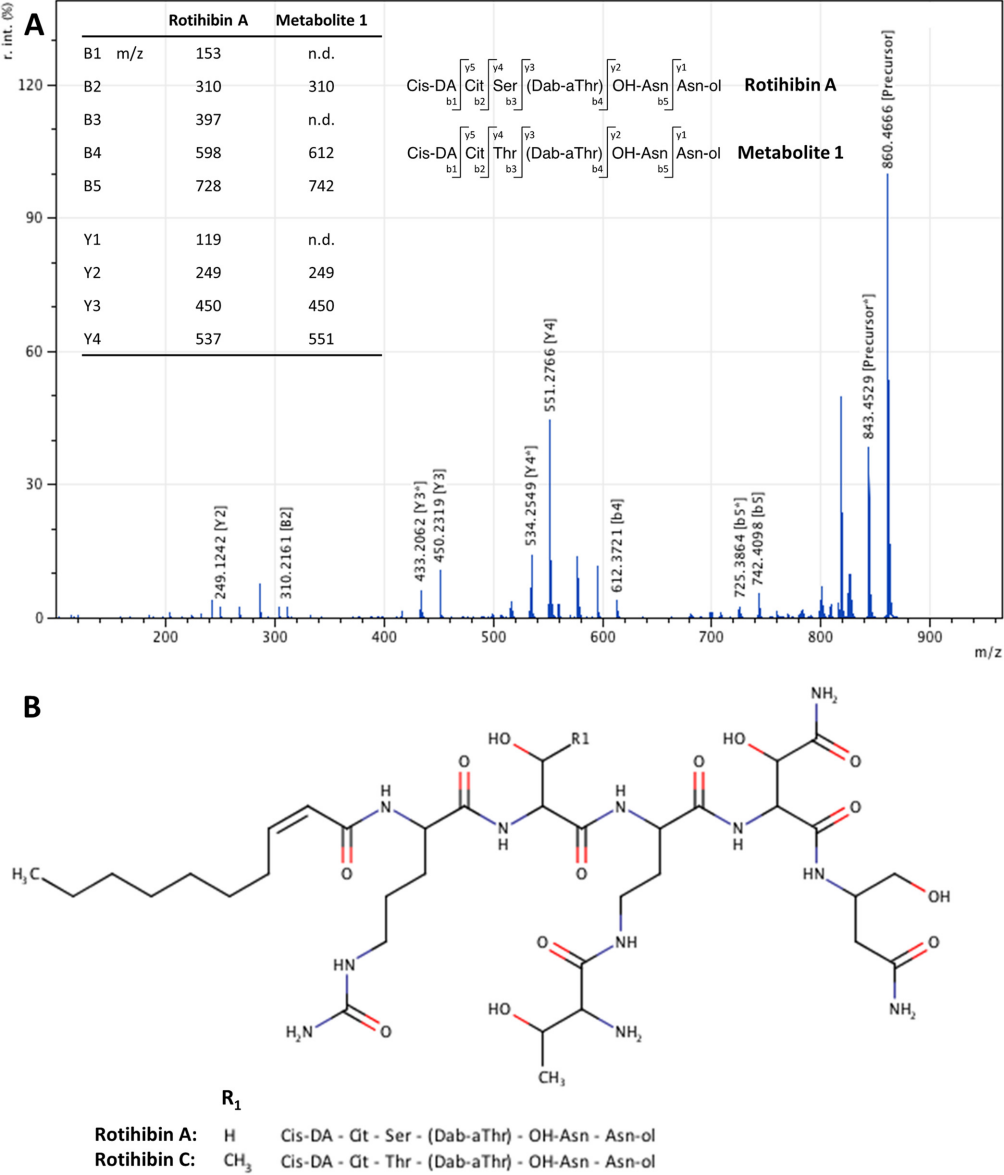
(A) MS/MS spectrum of the metabolite eluting after 5.52 min as displayed in Fig. 1. The fragment ions of this compound, designated rotihibin C (metabolite 1), are compared with those from rotihibin A, displaying a difference only in the NRP backbone (Ser→Thr). n.d., not detected. (B) Proposed structure of rotihibin C compared to the previously identified rotihibin A.

The ESI spectrum of the fraction eluting at 5.52 min displayed a major peak at *m/z* 860.47, while the spectrum of the fraction eluting at 5.75 min displayed a peak at *m/z* 874.47, which is also present in the 5.52-min sample (see Fig. S1 in the supplemental material). Both molecular ions were selected for tandem MS (MS/MS) analysis. Putative daughter *b*-ions at *m/z* 742 [M + H − 118]^+^, 612 [M + H − 118 − 130]^+^, and 310 [M + H − 118 − 130 − 302]^+^ and *y*-ions at *m/z* 551 [M + H − 309]^+^, 450 [M + H − 309 − 101]^+^, and 249 [M + H − 309 − 101 − 201]^+^ were observed in ESI-MS/MS analyses of the precursor ion at *m/z* 860.47 (Fig. 2). The bioinformatics tool Insilico Peptidic Natural Products Dereplicator was used to dereplicate the structure through database searching of mass spectra (27). The mass spectrum of rotihibin A, a nonribosomal peptide (NRP) functional as a plant growth regulator in Streptomyces graminofaciens, was found to have the best fit to our data. However, the spectrum of our newly detected compound from *S. scabies* indicated the presence of a threonine in the NRP backbone instead of a serine (as in rotihibin A) (Fig. 2A) (25). Indeed, the neutral losses of 118, 130, 302, 309, 101, and 201 mass units correspond to the masses of asparaginol (Asn-ol); hydroxy-asparagine (OH-Asn); (*allo*)-threonine (aThr), 2,4-diamino butyric acid (Dab), and threonine (Thr); 2-*cis*-decenoic acid (*cis*-DA) and citrulline (Cit); threonine (Thr); and aThr and 2,4-diamino butyric acid, respectively (Fig. 2B). Previously, Halder et al. established a chemical synthesis pathway of rotihibin A and structural analogues (26). In their structural analogue RotA-D3, the serine residue at position 2 of the amino acid chain was replaced by alanine. This derivative turned out to be more active than rotihibin A, as shown by the overall more pronounced plant growth retardation in an A. thaliana bioassay (26). Interestingly, our data suggest that *S. scabies* RL-34 would produce a natural rotihibin variant that is modified at the same position.

Fragmentation of the ion at *m/z* 874.47 displayed the same fragment ions, except for the daughter ions containing the acyl chain (*m/z* +14), which could be explained by a longer/branched acyl chain. For further description, we named the compound at *m/z* 860 rotihibin C and the compound at *m/z* 874 rotihibin D. For each of the compounds, we also see a component that is 2 Da smaller, which we assume to be due to desaturation. We also see a minor component at *m/z* 844/846, which, according to the MS/MS spectrum (data not shown), is a similar compound with a shorter fatty acyl chain.

**FIG 3:**
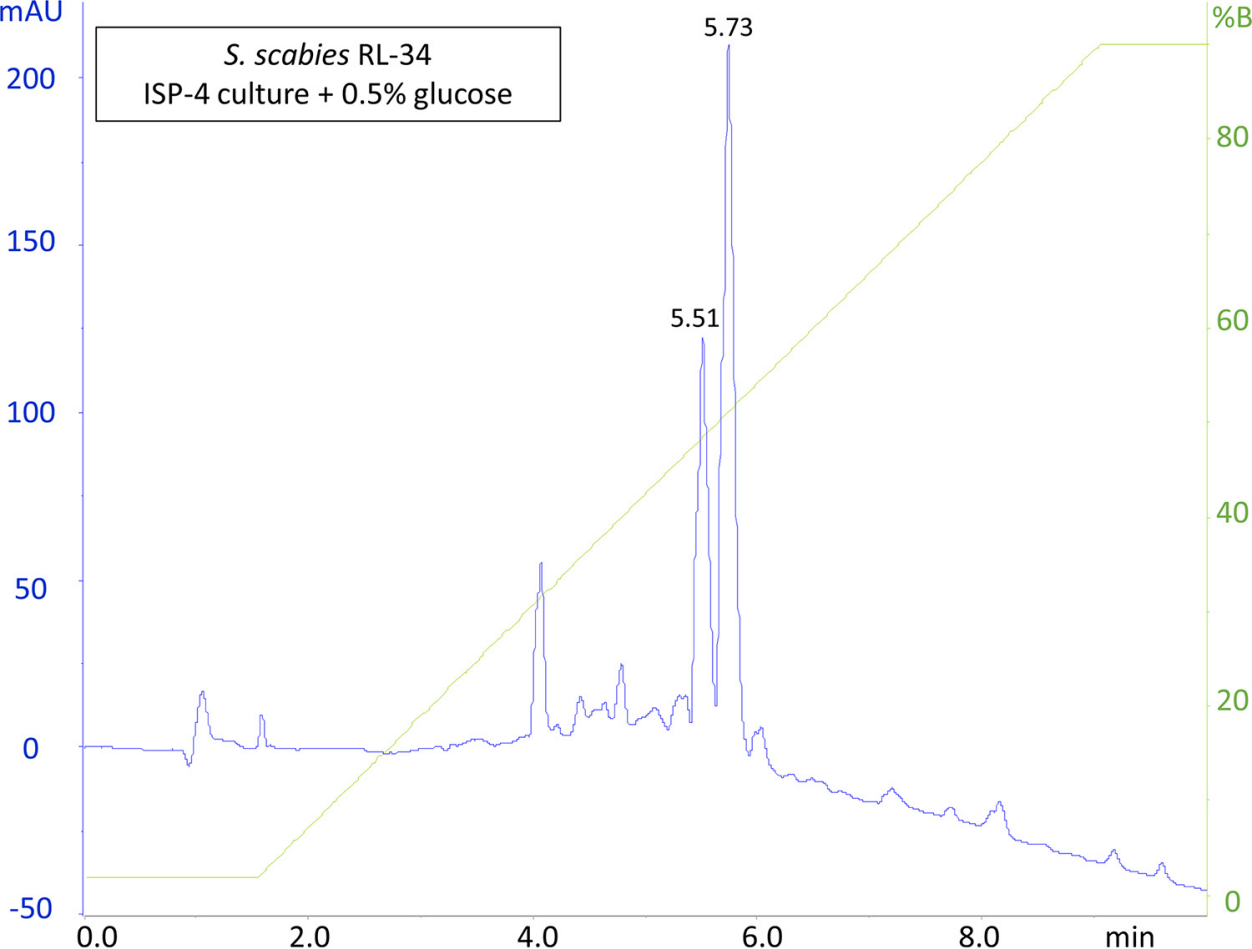
Effect of glucose on the secreted metabolome of *Streptomyces scabies* RL-34. An HPLC run was performed on *n*-butanol extracts of *Streptomyces scabies* RL-34 ISP-4 cultures grown in the presence of glucose. mAU, milli-absorbance unit.

Glucose is known to suppress thaxtomin A production in *Streptomyces scabies* and Streptomyces acidiscabies (28, 29). The effect of glucose, a breakdown product of cellobiose, on the production of the rotihibin analogues was tested. Indeed, the peak eluting at 6.1 min, corresponding to thaxtomin A, disappeared in the HPLC profile of the *S. scabies* RL-34 cultures grown in the presence of glucose. In contrast, compounds eluting at 5.52 and 5.75 min were still present (Fig. 3), and they were confirmed by direct-infusion HR-ESI-MS/MS to be rotihibins C and D (data not shown). This is a clear indication that cellobiose is not the trigger for rotihibin production in *S. scabies* RL-34 as it is for thaxtomin A.

Finally, we verified whether rotihibins C and D are also produced by other *S. scabies* strains. We performed similar extraction and purification methods on extracellular medium from the hypervirulent *S. scabies* 87-22 strain (12). The chromatograms and ESI spectra of these components were identical, indicating that this strain is also able to produce the same compounds (data not shown).

**FIG 4:**
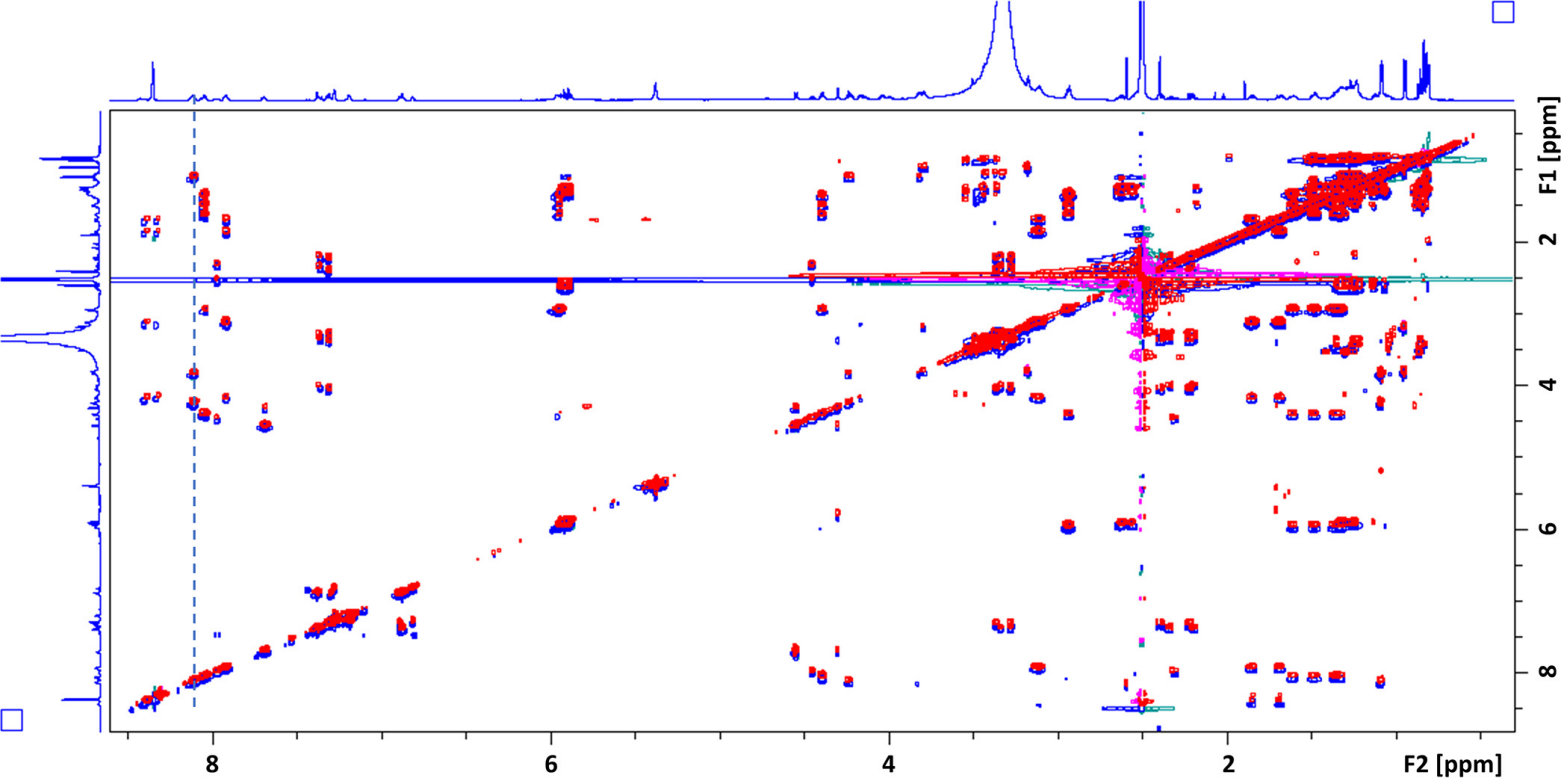
2D ^1^H-^1^H TOCSY spectra used for the analysis of the rotihibin C and D structures. The 2D TOCSY spectra of rotihibins C (blue) and D (red) are shown overlaid, with a slight offset of one with respect to the other to clearly show the identity of the correlation patterns for the peptide chains in both compounds. The dashed line identifies the correlation belonging to the threonine residue.

To confirm the structure proposed from the MS data, HPLC-purified rotihibins C and D were characterized using ^1^H nuclear magnetic resonance (NMR). Superposition of the two-dimensional (2D) total correlation spectroscopy (TOCSY) spectra of both compounds as depicted in Fig. 4 clearly indicates the presence of identical correlation patterns. In addition, analysis of the various spin systems in the TOCSY spectrum showed good agreement with the amino acid residues expected from the MS/MS analysis of the peptide chains of rotihibins C and D. Specifically, it agrees with our suggestion that a threonine in rotihibin C replaces the serine observed in rotihibin A. Since the NMR spectra of purified rotihibins C and D did not reveal structural differences between the peptide chains, the NMR analysis also supports that the 14-Da difference in mass likely results from an extra methylene group for the acyl chain in rotihibin D; however, the spectral overlap between the many CH_2_ units does not allow us to clearly establish acyl chain length using NMR.

### Identification of the biosynthetic gene cluster responsible for rotihibin production.

**FIG 5:**
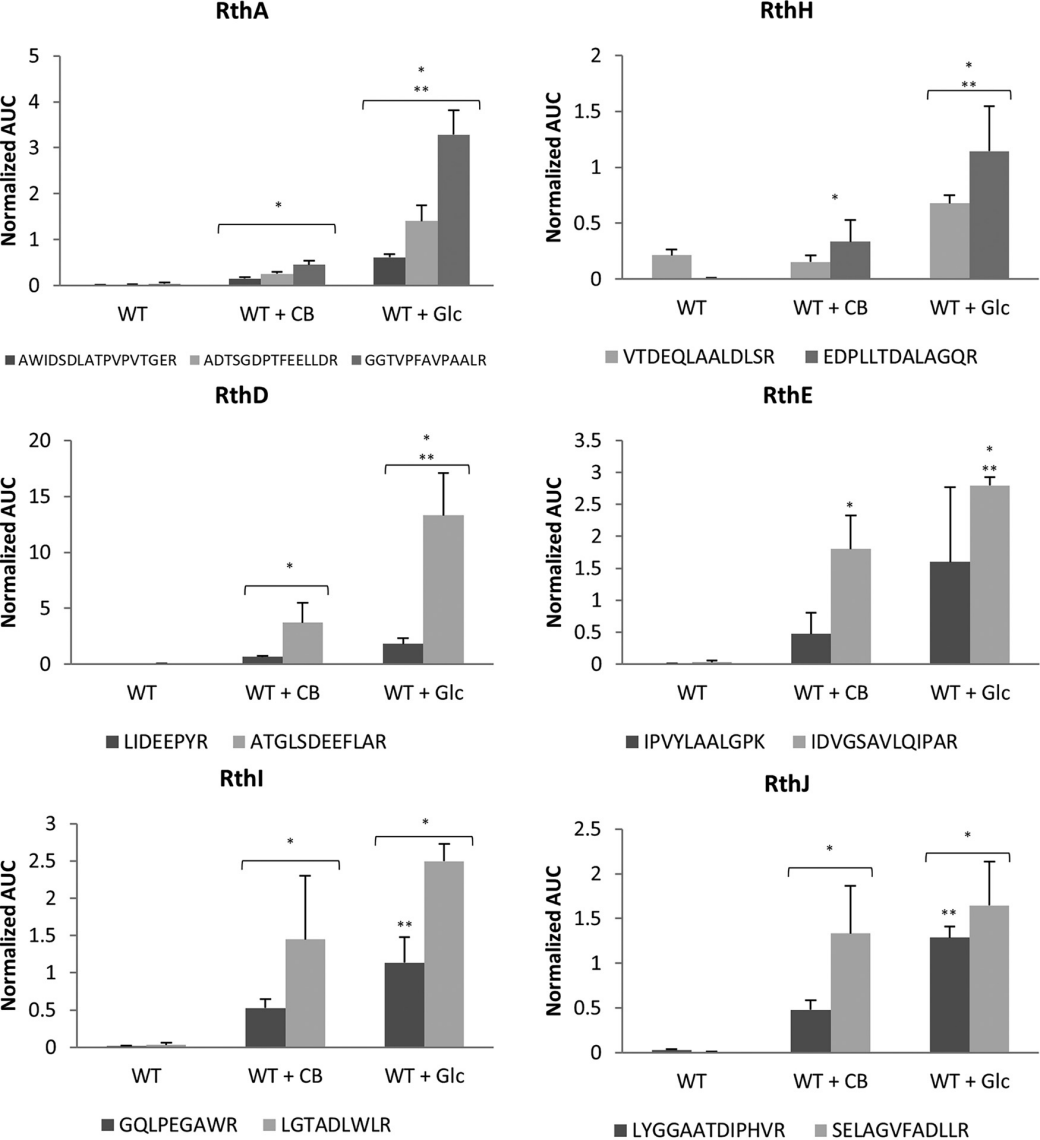
Relative abundances of proteins as part of nonribosomal peptide machinery in response to cellobiose (CB) (Glc_2_) or glucose (Glc) supply determined by targeted proteomics (LC-MRM). The plot displays the average area under the curve for each peptide used as a marker for the different proteins, normalized to the spike-in standard BSA. These results show significant normalized quantitative peptide abundances (*P* < 0.05) compared to the wild-type (WT) strain grown in ISP-4 without supplementary oligosaccharides (*) and with cellobiose (**). A statistical two-sided Student *t* test (homoscedastic) was performed. The error bars plot the standard deviations (SD) from three biological replicates. Note that we already adopted the rotihibin gene cluster annotation (see the text and Table 1).

Apart from the thaxtomin biosynthetic gene cluster (BGC), *S. scabies* also possesses four other NRP synthase (NRPS)-type BGCs; two of them are cryptic, that is, BGCs for which no biomolecule has yet been associated with the genetic material. Proteins associated with a cryptic NRPS-type BGC (from *scab_3221* to *scab_3351*) (see details below) were found in a previous proteomic experiment (15). We investigated the possible relationship between this BGC and rotihibin production by targeted proteomics using multiple-reaction monitoring (MRM). The proteotypic peptides of SCAB_3241 (LIDEEPYR and ATGLSDEEFLAR), SCAB_3251 (IPVYLAALGPK and IDVGSAVLQIPAR), SCAB_3281 (VTDEQLAALDLSR and EDPLLTDALAGQR), SCAB_3291 (GQLPEGAWR and LGTADLWLR), SCAB_3301 (LYGGAATDIPHVR and SELAGVFADLLR), and SCAB_3321 (AWIDSDLATPVPVTGER, ADTSGDPTFEELLDR, and GGTVPFAVPAALR) were used to evaluate the protein levels. Figure 5 shows the positive correlation between the presence of either glucose or cellobiose and the production of the selected proteins, thereby providing the first evidence that rotihibin production and this cryptic BGC might be linked. Interestingly, the inactivation of the cellulose utilization regulator *cebR* had no effect on the production of enzymes for this cluster or even instead had the reverse effect for the *scab_3221* gene product (15). This indicates that the effect of cellobiose on the expression of the NRPS gene cluster is not dependent on *cebR* and that there is no coregulation with thaxtomin production.

We then performed a bioinformatic analysis of this gene cluster to investigate whether it effectively contains the information for enzyme machinery allowing rotihibin production. We first analyzed the cryptic gene cluster using antiSMASH (30). It resides on a 70.1-kb segment of the *S. scabies* genome with 51 open reading frames (ORFs). The region around the largest NRPS gene (*scab_3321*) was compared with all public genomes using PATRIC (31). This analysis confined the cluster to a 33-kb segment with 14 ORFs (*scab_3221* to *scab_3351*). The presence of a putative integrin protein (SCAB_3201) and a putative transposase (SCAB_3371) at the borders of this cluster is evidence for the possible mobilization of this gene cluster.

**FIG 6:**
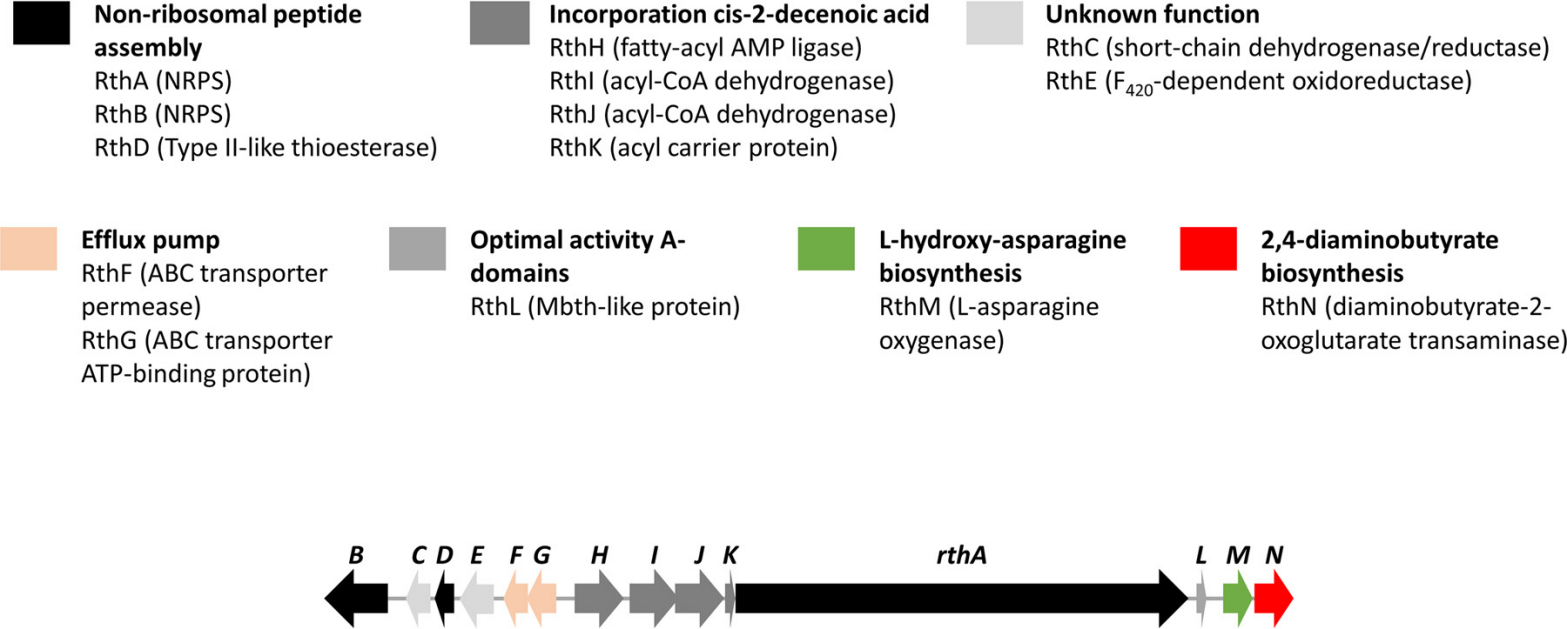
The rotihibin gene cluster proposed to be responsible for the production and secretion of rotihibins.

The sequences of these 14 ORFs were individually submitted to a BLAST search against the UniProtKB/Swiss-Prot database, and putative functions derived from this search are listed in Table 1 and Fig. 6. A preliminary analysis of these functions allowed us to conclude that this gene cluster contains the necessary information to produce the enzymes needed for the biosynthesis of rotihibins C and D, as outlined below. Therefore, this cluster is further referred to as the rotihibin (*rth*) gene cluster.

**TABLE 1:** Functions of proteins encoded by the rotihibin biosynthetic gene cluster

Gene	Protein	Predicted function
SCAB_3221	RthB	Unimodular nonribosomal peptide synthetase
SCAB_3231	RthC	Short-chain dehydrogenase/reductase
SCAB_3241	RthD	Type II-like thioesterase enzyme
SCAB_3251	RthE	F_420_-dependent oxidoreductase
SCAB_3261	RthF	ABC transporter permease
SCAB_3271	RthG	ABC transporter ATP-binding protein
SCAB_3281	RthH	Fatty acyl-AMP ligase
SCAB_3291	RthI	Acyl-CoA dehydrogenase
SCAB_3301	RthJ	Acyl-CoA dehydrogenase
SCAB_3311	RthK	Acyl carrier protein
SCAB_3321	RthA	Pentamodular nonribosomal peptide synthetase
SCAB_3331	RthL	MbtH-like protein
SCAB_3341	RthM	l-Asparagine oxygenase
SCAB_3351	RthN	Diaminobutyrate-2-oxoglutarate transaminase

### Analysis of the genes involved in the rotihibin BGC. (i) NRPS genes for peptide chain assembly.

**FIG 7:**
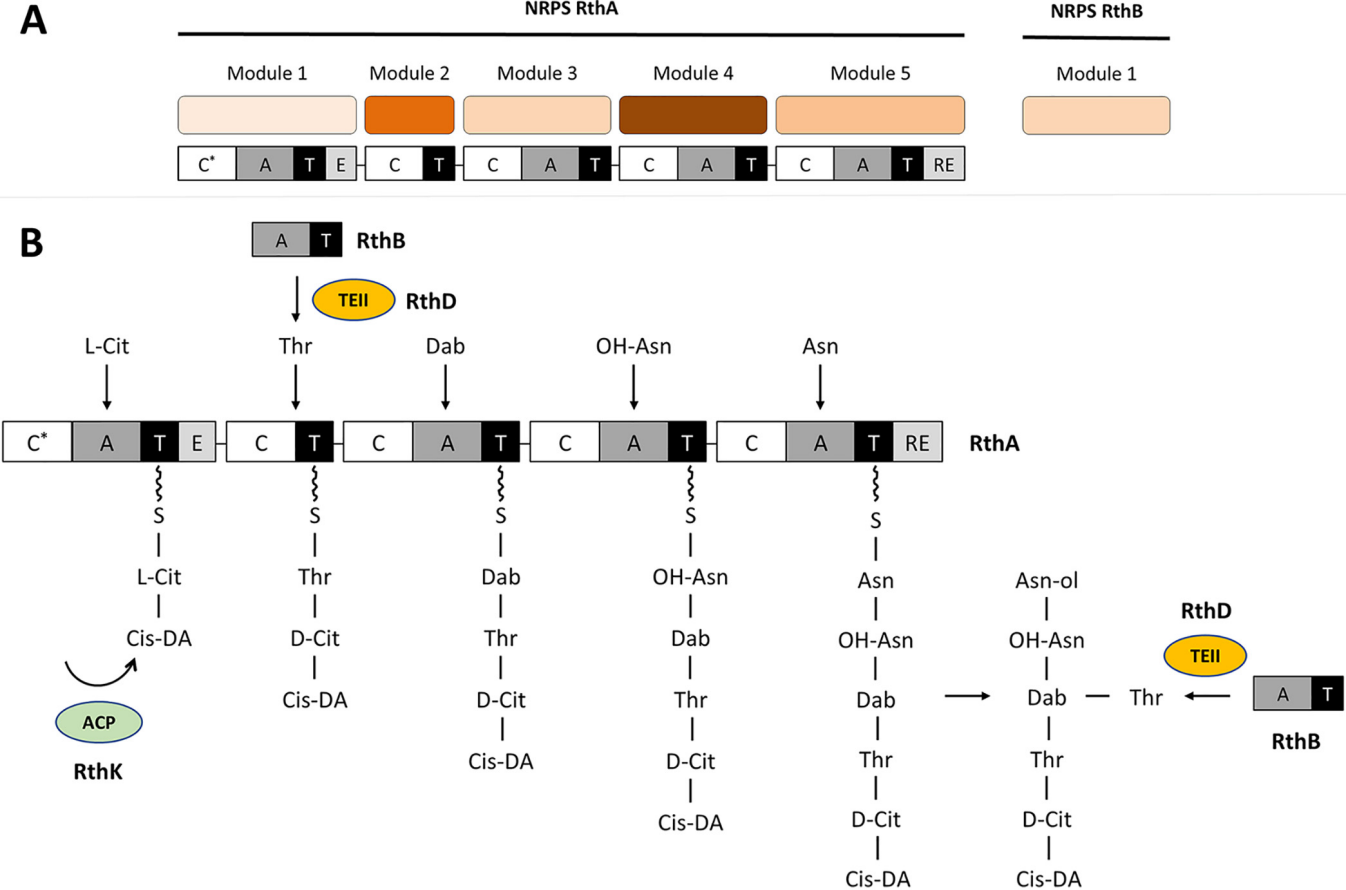
(A) Organization of the NRPSs in the rotihibin gene cluster. RthA contains five modules, while RthB has only one module. C, condensation; A, adenylation; T, thiolation; E, epimerization; RE, reduction. (B) Proposed biosynthetic pathway and modular organization of the NRPS for rotihibin biosynthesis. RthA is responsible for the NRP backbone. Cis-DA, *cis*-2-decenoic acid; Cit, citrulline; Dab, (2,4)-di-amino butyric acid; C, condensation domain; A, adenylation domain; T, thiolation; E, epimerization domain; RE, reductase domain; ACP, acyl carrier protein.

Two NRPSs, RthA and RthB, are present in this cryptic gene cluster. RthA is predicted to contain 5 modules, while RthB contains a single module for the incorporation of amino acids in a peptide chain (Fig. 7A). Different tools were used to predict their NRPS adenylation domain (A domain) specificity (Table 2), and from these results, we propose a biosynthetic path for the production of the rotihibin peptide chain (Fig. 7B). Module 1 of RthA was predicted to be specific for Glu or Ser, depending on the tool used. However, we believe that module 1 is responsible for the incorporation of citrulline, the first amino acid of the peptide: the signature sequence for an A domain that could be specific for citrulline is possibly not included in the prediction tools. The A domain was missing from module 2, while (*allo*)-threonine should be built into the rotihibin backbone. RthB is the best candidate to introduce threonine into the rotihibin peptide sequence, as its A domain is predicted to be specific for threonine. This phenomenon was previously described in the enduracin biosynthetic gene cluster from Streptomyces fungicidicus. Another NRPS activates and transfers l-*allo*-threonine to the module with the missing A domain (32). RthD, a type II thioesterase-like (TEII) enzyme, is believed to assist in the shuttling of the activated aThr between the stand-alone A-T didomain module RthB and the A-less C-T module of RthA, as described previously for WS9326A biosynthesis (33). A serine residue was predicted in module 3, but (2,4)-di-amino butyric acid was observed. The prediction of the two last modules to introduce asparagine residues is in line with the rotihibin structure having two asparagine-like residues at its C terminus. Module 5 has a reductase (RE) domain. This domain is known to reduce the peptidyl thioester into its corresponding alcohol, which explains the presence of l-asparaginol (34). This also explains why rotihibin does not form a cyclic structure like many other NRPS-derived lipopeptides.

**TABLE 2:** NRPS adenylation domain specificity prediction^*a*^

Module	Domain specificity predicted by method								Corresponding amino acid
	LSI based		NRPSsp		PKS/NRPS		SEQL-NRPS		
	Pred	Score (%)	Pred	Score (HMMER bit)	Pred	Score (bits)	Pred	Prob	
RthA									
1	Glu	0.595	Ser	661.3	Glu	16.5	Ser	0.485	Lipo-d-Cit
2									l-*allo*-Thr
3	Arg	0.528	Ser	568.5	Ser	16.9	Ser	0.474	l-Dab
4	Asn	0.934	Asn	486.6	Asn	16.5	Asn	0.615	l-Asn-OH
5	Asn	0.700	Asn	450.4	Asn	18.1	Orn	0.463	l-Asn

RthB									
1	Thr	0.955	Thr	651.9	Thr	19.2	Thr	0.469	Thr

a The specificities of the A domains of the different modules in RthA and RthB were predicted using different software tools. Pred, predicted amino acid; Prob, probability.

Potentially, RthB has a dual function by transferring l-*allo-*threonine to module 2 and building an additional l-*allo-*threonine into the NRP backbone by forming an *iso*-peptide bond with (2,4)-di-amino butyric acid. This was also described for viomycin and capreomycin, where one A domain is proposed to function twice, to acetylate not only the T domain within its own module but also the T domain of another module (35, 36).

The stereochemistry of the different amino acids of rotihibin A was previously determined by Marfey’s method (23). 2,4-Diamino butyric acid (Dab), aThr, Asn-ol, and hydroxy-asparagine (OH-Asn) were proven to be in the l-form, and the citrulline (Cit) residue was proven to be in the d-form. The epimerization (E) domain in module 1 confirms the results of this experiment, as it is known to convert amino acids between the l- and d-isomers. Many NRPSs require MbtH-like proteins (MLPs) for the proper folding and activity of the NRPS. This protein is essential for the biosynthesis of, for example, pyoverdine (37), vancomycin (38), coelichelin, and the calcium-dependent antibiotic (CDA) (39). *rthL*, encoding an MbtH-like protein, is probably necessary for the production of the rotihibins. Li et al. showed that RthL can functionally replace TxtH in the thaxtomin biosynthetic pathway, demonstrating that MLPs from different pathways are able to complement each other (40).

### (ii) Biosynthesis of nonproteinogenic amino acids.

Three nonproteinogenic amino acids are incorporated into the rotihibin nonribosomal peptide backbone: Cit, Dab, and OH-Asn. *rthM* encodes an l-asparagine oxygenase (AsnO), which we propose to be involved in converting Asn to OH-Asn. AsnO was previously found in the biosynthetic gene cluster of CDA (41) and A54145 (42). Finally, the unusual amino acid 2,4-diamino butyric acid is produced from aspartate β-semialdehyde by the enzyme diaminobutyrate-2-oxoglutarate transaminase (43, 44). *rthN* encodes a homologue of this enzyme and is proposed to be responsible for Dab biosynthesis, which is finally incorporated into rotihibin. There is no specific gene that could be responsible for citrulline synthesis, but this is an intermediate of arginine biosynthesis that could be sufficiently available.

### (iii) Formation and attachment of the fatty acid tail.

**FIG 8:**
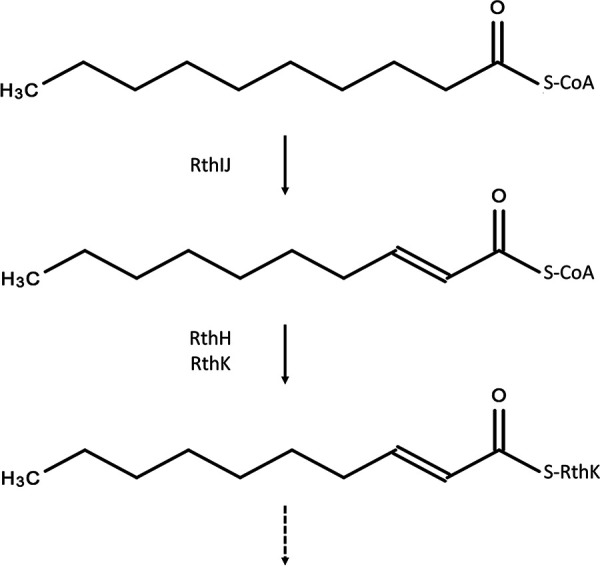
Proposed activation and transfer of *cis*-2-decenoic acid (*cis*-DA).

Numerous lipopeptides, synthesized by NRPS complexes, have already been described in *Streptomyces* species, for example, A54145 (42), CDA (45), daptomycin (46), and enduracidin (32). These clusters contain genes whose products act together to acylate the first amino acid. Bioinformatic analysis of the *rth* gene cluster revealed the presence of a gene encoding an acyl carrier protein (*rthK*), which is immediately flanking *rthA*. This protein is proposed to transfer medium-chain fatty acids to the N-terminal domain of the Cit-incorporating module (Fig. 8). Fatty acyl-AMP ligases are enzymes establishing the cross talk between fatty acid synthases and NRPSs or polyketide synthetases (PKSs). They initiate the biosynthesis of lipopeptides by the activation of a fatty acyl residue and occur with a high incidence in putative lipopeptide NRPS/PKS clusters (47). RthH is supposed to recruit a fatty acid and transfer it to the acyl carrier protein RthK. The fatty acid side chain lengths and/or degrees of saturation are the only differences between the rotihibin C and D analogues. The desaturation could be explained by the presence of two acyl-CoA dehydrogenases (RthI and RthJ), similar to the situation in the ramoplanin biosynthetic gene cluster (48). One is expected to introduce the first double bond, while the second additional dehydrogenation is possibly not essential. The minor amounts of other lipopeptides identified via MS, which differ by only −2 Da, could be explained this way (Fig. S1). This phenomenon was previously described in acyl-desferrioxamines of Streptomyces coelicolor (49).

### (iv) Self-resistance genes.

During antibiotic production, transporter and transporter-associated proteins are important for the import of effector molecules, self-resistance, and guiding/exporting the antibiotic to the extracellular environment. *rthF* and *rthG* are predicted to act as the rotihibin-exporting machinery as they encode an ABC-type permease and an ABC transporter ATP-binding protein, respectively, a combination often found in antibiotic biosynthetic gene clusters. RthF shows 47% similarity with the daunorubicin/doxorubicin resistance ABC transporter permease protein DrrB, while RthG shows 61% similarity with the daunorubicin/doxorubicin resistance ATP-binding protein DrrA in Streptomyces peucetius. This DrrAB efflux system in S. peucetius has been shown to be a multidrug transporter with broad specificity (50). RthFG possibly has a similar role in the self-resistance mechanism of *S. scabies*.

### (v) Other genes within the *rth* cluster.

The BGC for rotihibin in *S. scabies* houses a number of genes not directly associated with rotihibin assembly. The protein product of *rthE* is predicted to be an F_420_-dependent oxidoreductase, and *rthC* encodes a short-chain dehydrogenase/reductase. These two enzymes are, for example, directly involved in the production of coronafacoyl phytotoxins in *S. scabies* (51). The impact of the inactivation of these genes on rotihibin biosynthesis has to be studied to reveal their exact role.

### (vi) Inactivation of *rthB* abolishes rotihibin production.

**FIG 9:**
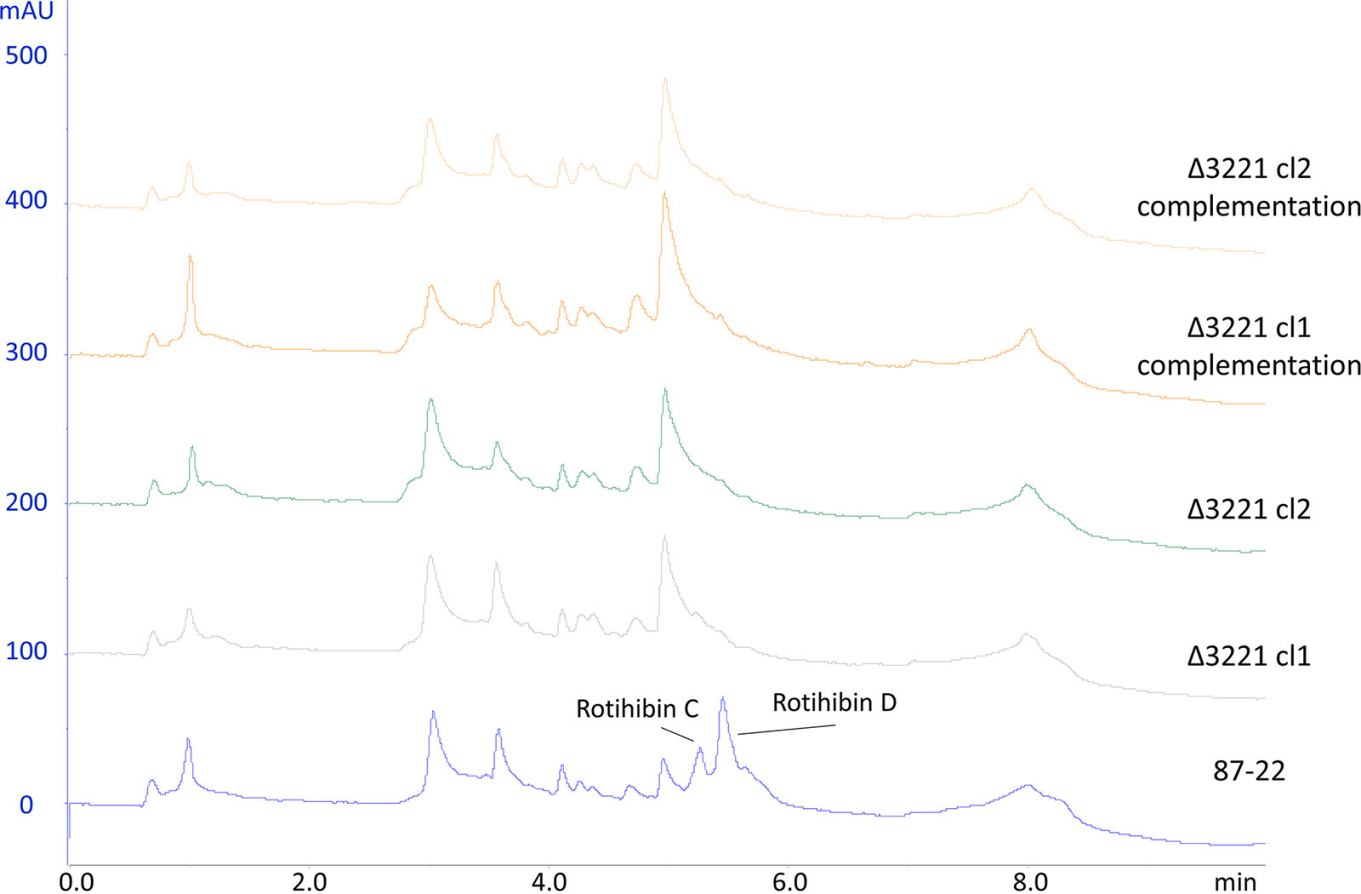
Inactivation of the nonribosomal peptide gene *rthB.* Shown are data from HPLC analysis of rotihibin production by *Streptomyces scabies* 87-22 wild-type, mutant, and complementation strains. mAU, milli-absorbance unit.

To ascertain whether the *rth* cluster is responsible for the production of rotihibins, the *scab_3221* gene was disrupted and replaced in *S. scabies* 87-22 by an apramycin resistance cassette (11, 13, 29). HPLC analysis and targeted metabolomics on *n*-butanol extracts of the Δ*rthB* strain confirmed the absence of rotihibins (Fig. 9). These data support the evidence that the *rth* BGC is indeed responsible for rotihibin production. Simple complementation of the Δ*rthB* strain with an integrative plasmid harboring an intact copy of *rthB* with its own promoter did not restore the production of rotihibins, which could be attributed to polar effects after the removal of the *rthB* gene and/or the insertion of the new copy of *rthB* in a region of the chromosome not optimal for its expression.

### Biological activity of rotihibins C and D.

**FIG 10:**
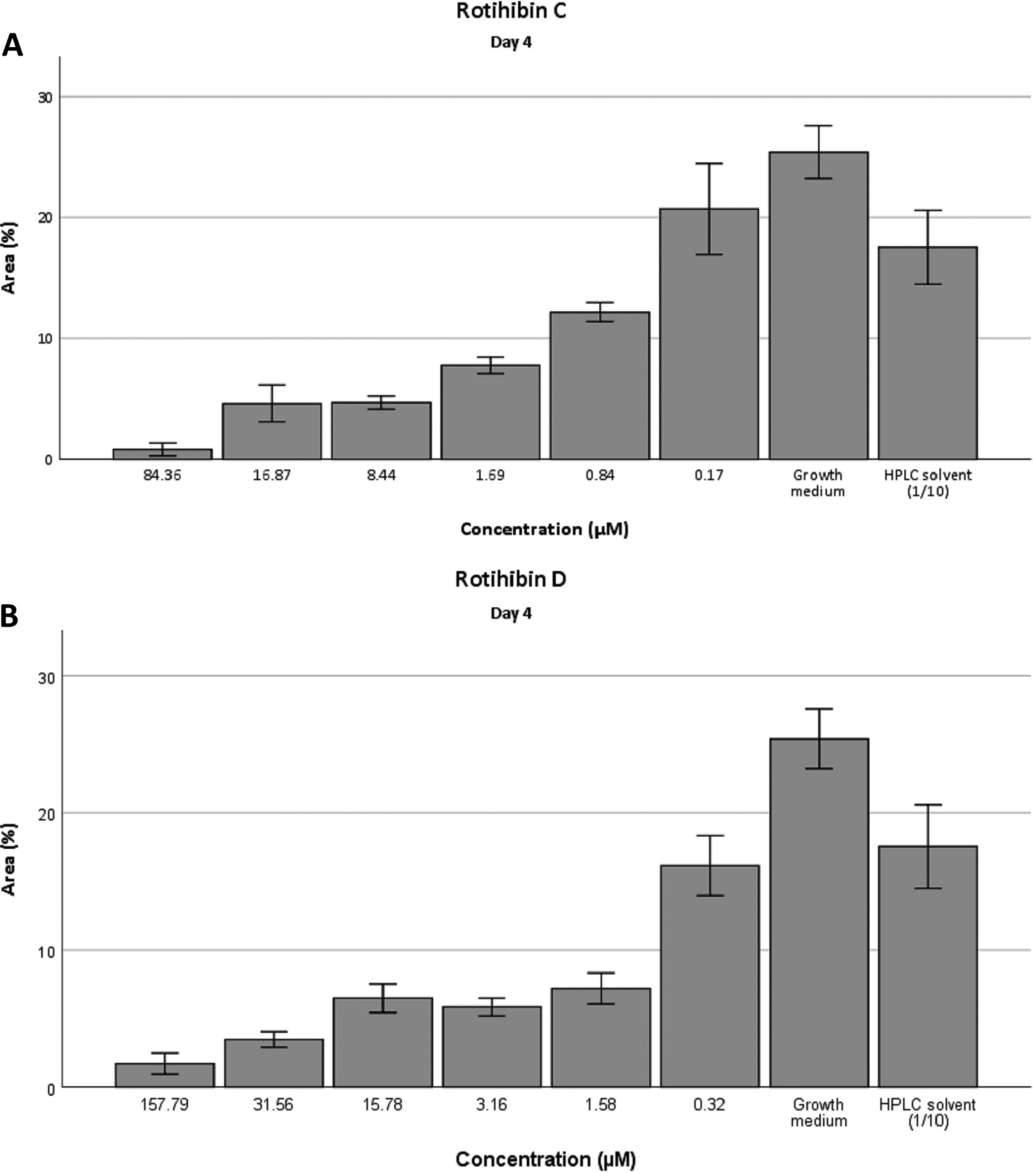
Surface area measurements of *Lemna minor* treated with rotihibins. The plant growth-inhibitory activity of rotihibin C (A) and rotihibin D (B) was analyzed after 4 days compared to the control. Data are given as means from four replicates ± standard deviations (SD).

Since rotihibin A has a plant growth-inhibitory effect, we tested whether the new rotihibins C and D display similar properties. After 4 days of exposure to concentrations ranging from 0.84 to 84.4 μM rotihibin C and from 0.3 to 157.8 μM rotihibin D, the growth of duckweed (*Lemna minor* L.), especially the fronds, was suppressed compared to that in the control group (a neighboring HPLC fraction that did not display UV absorption) (Fig. 10).

**FIG 11:**
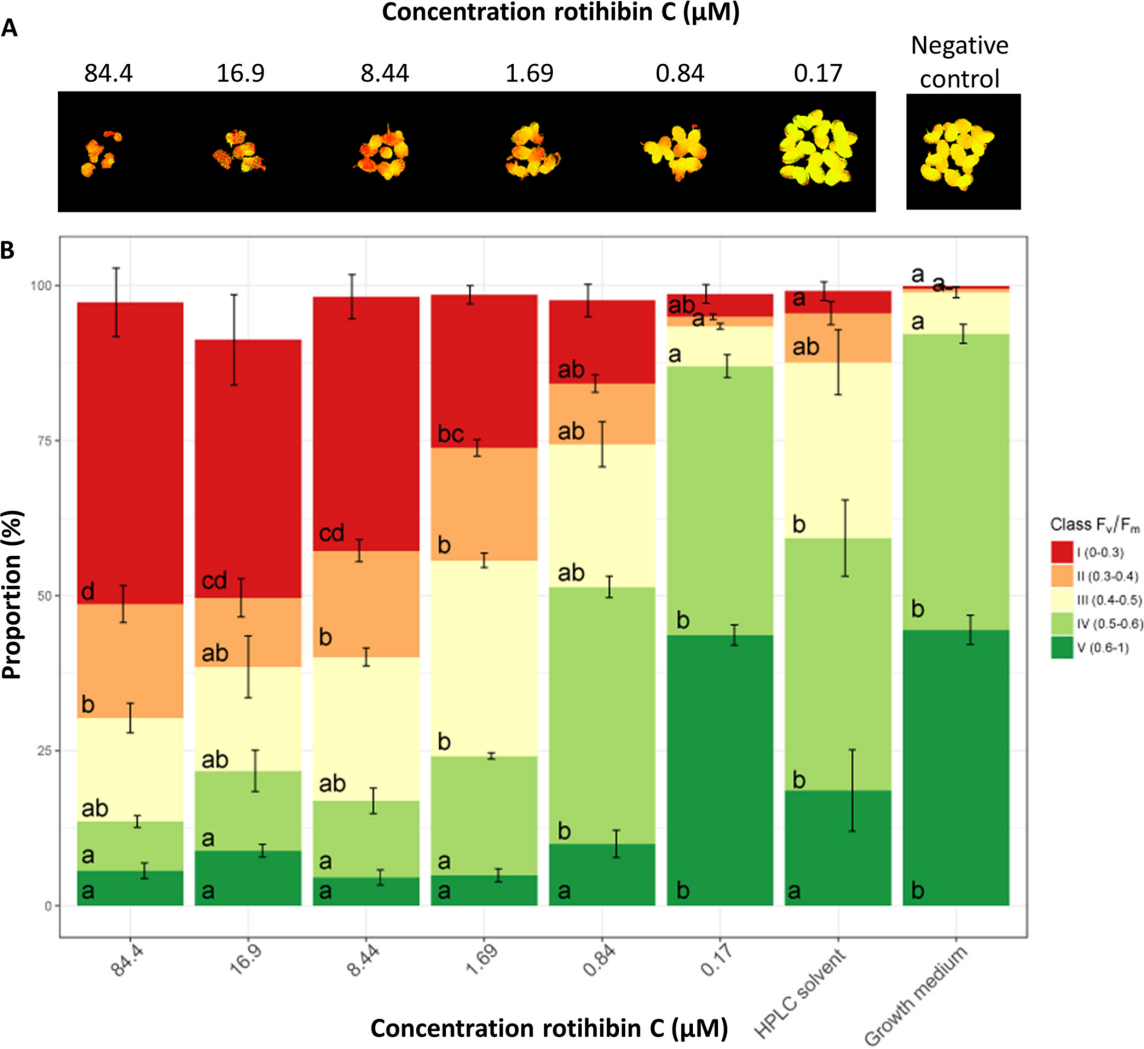
Effect of rotihibin C on the maximal photochemistry efficiency of photosystem II of *Lemna minor*. (A) Chlorophyll fluorescence of *Lemna minor* fronds. Data from one out of four replicates are shown. (B) The *F*_V_/*F*_M_ values showed a decreasing trend with increasing rotihibin C concentrations. Data processing and statistical analyses were performed using Welch’s *t* test and Tukey’s *post hoc* test.

**FIG 12:**
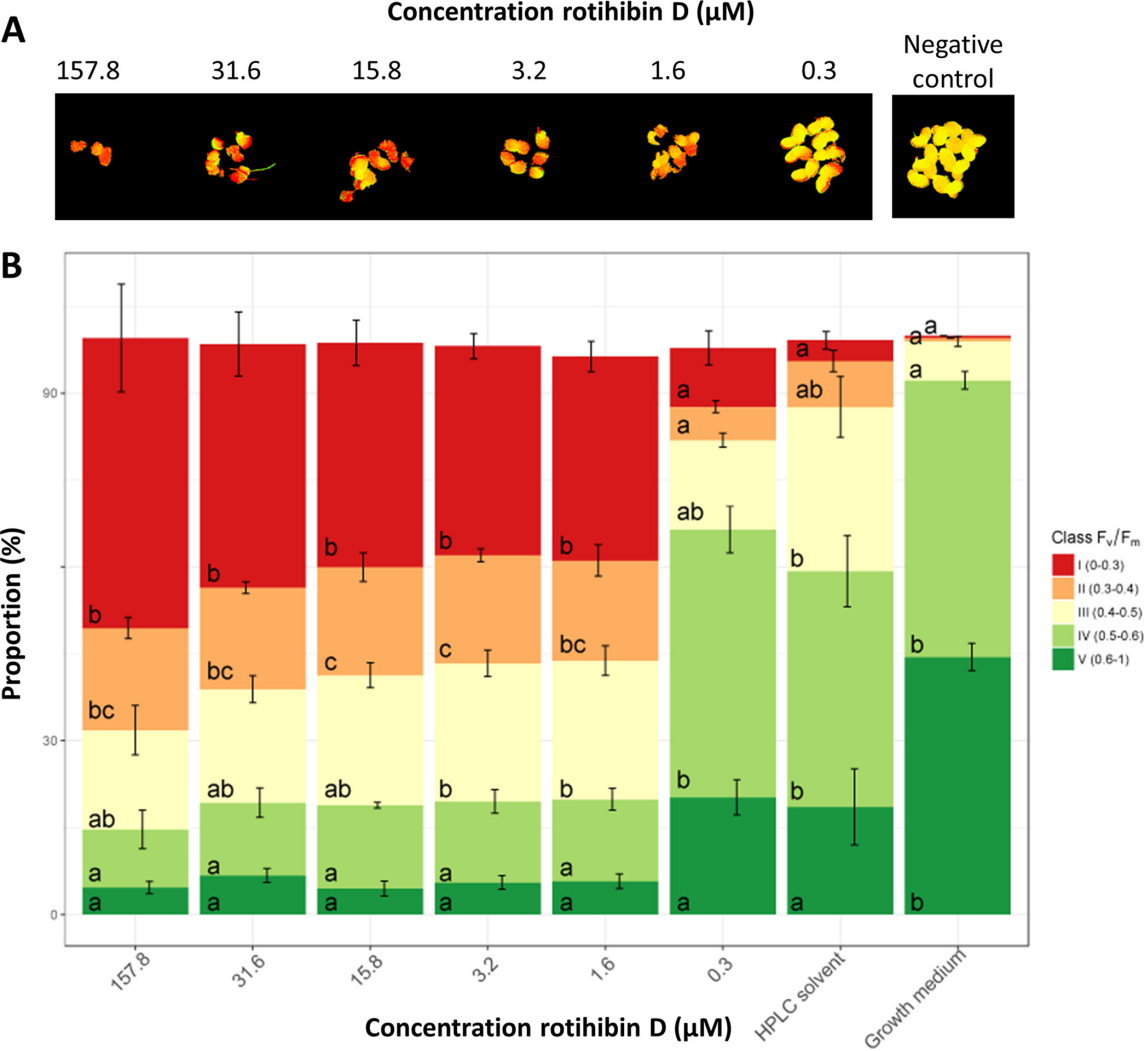
Effect of rotihibin D on the maximal photochemistry efficiency of photosystem II of *Lemna minor*. (A) Chlorophyll fluorescence of *Lemna minor* fronds. Data from one out of four replicates are shown. (B) The *F*_V_/*F*_M_ values showed a decreasing trend with increasing rotihibin D concentrations. Data processing and statistical analyses were performed using Welch’s *t* test and Tukey’s *post hoc* test (B).

Additionally, we assessed the impact of rotihibins C and D on the photochemistry of photosystem II using the *F*_V_/*F*_M_ ratio as a proxy. In dark-adapted *F*_V_/*F*_M_ measurements, the minimal fluorescence (*F*_0_) is measured. Next, an intense light flash is used to close (reduce) all reaction centers and measure the maximum fluorescence, called *F*_M_. The difference of *F*_M_ − *F*_0_ is the *F*_V_ value. The *F*_V_/*F*_M_ ratio represents the maximum potential quantum efficiency of photosystem II. In general, the greater the plant stress, the fewer open reaction centers available, which results in a lowered *F*_V_/*F*_M_ ratio. The RGB and chlorophyll fluorescence images are depicted in Fig. S2 to S5 in the supplemental material. Using the *F*_V_/*F*_M_ ratio as a proxy, the maximal photochemistry efficiency of photosystem II showed a decreasing trend with increasing rotihibin concentrations in the nutrient medium, revealing that rotihibins affect the photosynthesis of L. minor (Fig. 11 and 12).

The increased surface area of *L. minor* treated with 0.17 μM rotihibin C compared to the HPLC solvent indicates a hormetic effect. Hormesis is a dose-response phenomenon where a low dose of a toxic component promotes plant growth, while at higher doses, inhibition of growth is observed.

**FIG 13:**
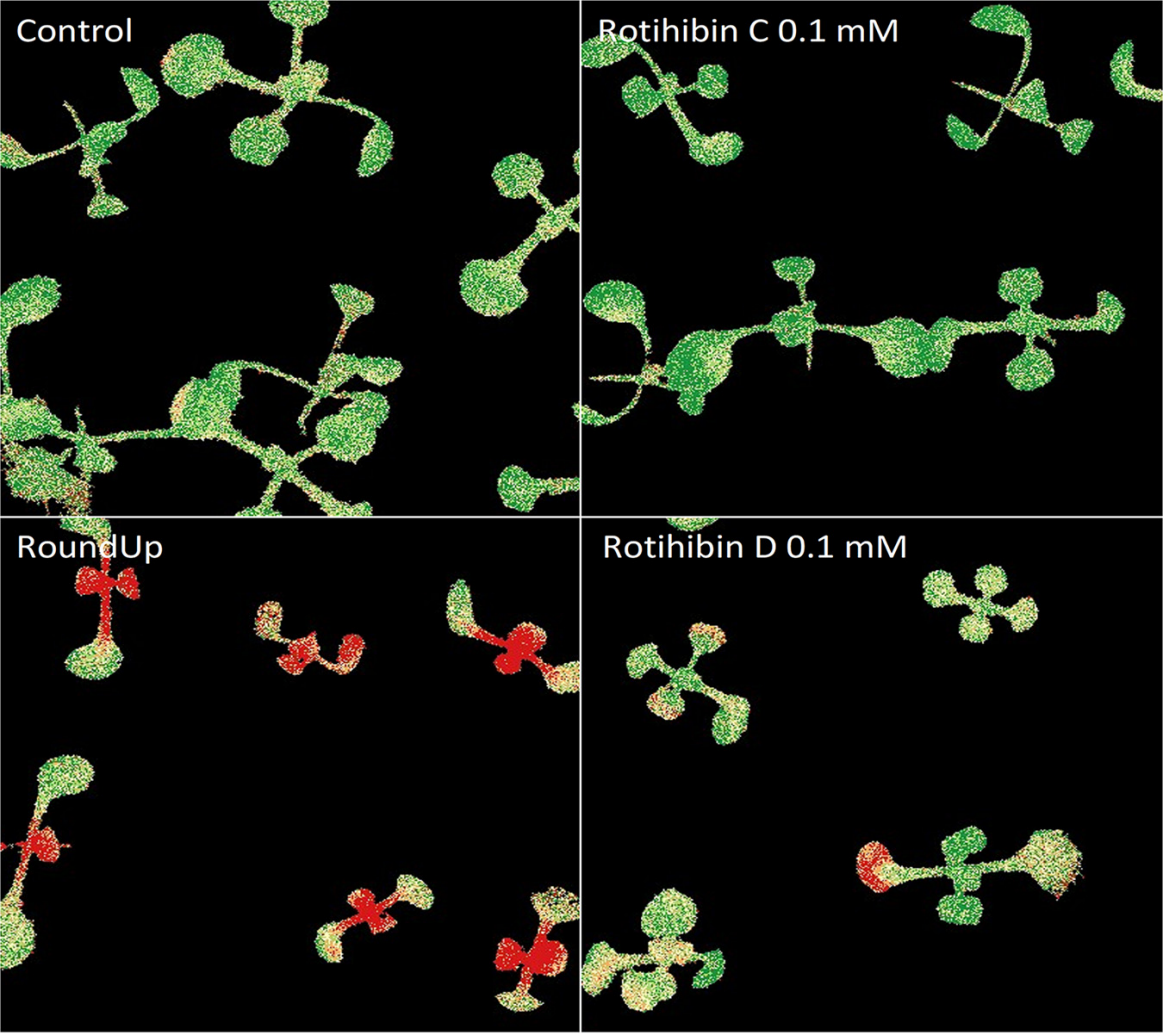
Effect of rotihibins C and D on Arabidopsis thaliana. The spraying of plants with 0.1 mM rotihibins C and D induced a negative effect on the growth and available reaction centers of photosystem II of Arabidopsis thaliana.

**FIG 14:**
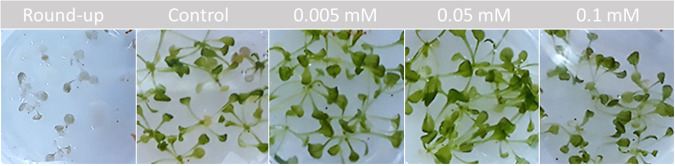
Hormetic effect of rotihibin D on Arabidopsis thaliana. At lower concentrations (0.05 mM and 0.005 mM), there is a beneficial effect of rotihibin D on plant growth, while at a higher concentration (0.1 mM), there is a toxic effect.

Ordinal analysis 96 h after application also showed an effect on the growth and maximal photochemistry efficiency of photosystem II (*F*_V_/*F*_M_) (52) of Arabidopsis thaliana plants treated with 0.1 mM rotihibins C and D (Fig. 13). Here, rotihibins were not added to the nutrient medium but were sprayed onto the plants; this explains the high concentration needed compared to the *L. minor* bioassay. Most herbicides are applied as water-based sprays, the easiest way to protect plant products for farmers. The positive control was a commercial glyphosate formulation (RoundUp Turbo) at a molar concentration of approximately 3 M. At lower concentrations of rotihibin D (0.05 mM and 0.005 mM), we observed growth promotion on *Arabidopsis* seedlings, which again reflects a hormetic effect (Fig. 14).

Recently, Halder et al. chemically synthesized rotihibin A and a number of variants (26). They provide evidence that rotihibin A targets the TOR (target of rapamycin) kinase (TORK) pathway, which explains the observed effects on plants. This highly conserved pathway is involved in the regulation of shoot and root development (53).

### Phylogenetic and evolutionary analyses of rotihibin biosynthesis.

The genomic region surrounding *rthA* was compared to all publicly available genomes using PATRIC, resulting in 24 *Actinobacteria* strains harboring a complete putative rotihibin BGC (Table 3). Rotihibin BGCs that split across more than one contig were not considered.

**TABLE 3:** Actinobacteria harboring a putative rotihibin biosynthetic gene cluster^*a*^

Organism	Source	Genbank genome assembly accession no.	Presence of rotihibin BGC form		
			E	O	X
Streptomyces scabiei 87-22		GCA_000091305.1			
Streptomyces scabiei NCPPB 4066	Solanum tuberosum, New York City, USA	GCA_000738715.1			
Streptomyces bottropensis FxanaA7		GCA_000958545.1			
Streptomyces bottropensis cf124		GCA_900114955.1			
Streptomyces galbus KCCM 41354	Tunisia	GCA_000772895.1			
Streptomyces stelliscabiei P3825		GCF_001189035.1			
Streptomyces stelliscabiei 1222.2		GCA_900215595.1			
Streptomyces ipomoeae B12321	Sweet potato storage root, Louisiana, USA	GCA_006547165.1			
Streptomyces caeruleatus NRRL B-24802	Tomato rhizosphere, Guangzhou, China	GCA_001514235.1			
Streptomyces ipomoeae 78-51	Bunkie, Louisiana, USA	GCA_006547175.1			
Streptomyces cinereoruber subsp. *fructofermentans* GY16	*Broussonetia papyrifera*, Hunan, Changsha city, China	GCA_009184865.1			
Streptomyces europaeiscabiei NCPPB 4086	Solanum tuberosum, Ontario, Guelph, Canada	GCA_000738695.1			
*Streptomyces geranii* A301	*Geranium carolinianum*, Emei mount, China	GCA_002954775.1			
Streptomyces diastatochromogenes CB02959	Putuo Moutain, Zhejian Province, China	GCA_002803155.1			
Streptomyces acidiscabies NCPPB 4445	Solanum tuberosum	GCA_001189015.1			
*Streptomyces ossamyceticus* JV178	Connecticut, USA	GCA_002761895.1			
Streptomyces rameus BK387, BGC 1		GCA_004342785.1			
Streptomyces rameus BK387, BGC 2		GCA_004342785.1			
Lechevalieria aerocolonigenes NRRLB-16140		GCA_000955955.1			
Streptomyces hygroscopicus subsp. *jinggangensis* 5008		GCA_000245355.1			
Streptomyces hygroscopicus subsp. *jinggangensis* TL01		GCA_000340845.1			
Streptomyces corchorusii DSM 40340	Bangladesh	GCA_001514055.1			
Streptomyces jiujiangensis NRRLS-31	El Salvador	GCA_000718775.1			
Streptomyces violaceorubidus NRRL B-16381		GCA_000717995.1			

a The *rth* gene clusters were classified regarding the presence of *rthE* (E form), *rthO* (O form), or neither (X form).

Strains of *S. scabies*, S. stelliscabiei, S. acidiscabies, S. europaeiscabiei, and *S. ipomoeae*, all known plant pathogens, are found to contain this rotihibin biosynthetic gene cluster and are thus predicted to produce rotihibin analogues. Other rotihibin BGC-containing species, in contrast, are known as plant-protecting bacteria: S. galbus has been shown to induce disease resistance by the accumulation of a phytoalexin in *A. thaliana*, thereby protecting it from the anthracnose disease caused by Colletotrichum higginsianum (54). S. caeruleatus is also a biocontrol agent by inhibiting the soybean pathogen Xanthomonas campestris pv. glycines (55). S. geranii sp. nov. was isolated from the root of Geranium carolinianum and shares the highest 16S rRNA sequence similarity to the plant pathogen *S. turgidiscabies* ATCC 700248 (56). S. hygroscopicus subsp. *jinggangensis* strains are known to produce the antibiotic validamycin (57), while S. corchorusii produces butalactin (58). The latter shows biocontrol and plant growth-promoting activities and potential as a biofertilizer agent for rice plants (59). The actinomycete Lechevalieria aerocolonigenes has been isolated from soil in Japan and is known to produce rebeccamycin with antitumor properties (60). Curiously, the cluster is found only in plant-associated species but is not restricted to plant pathogens. Rotihibin C and D production was experimentally verified in Streptomyces stelliscabiei NCPPB 4040 (data not shown).

**FIG 15:**
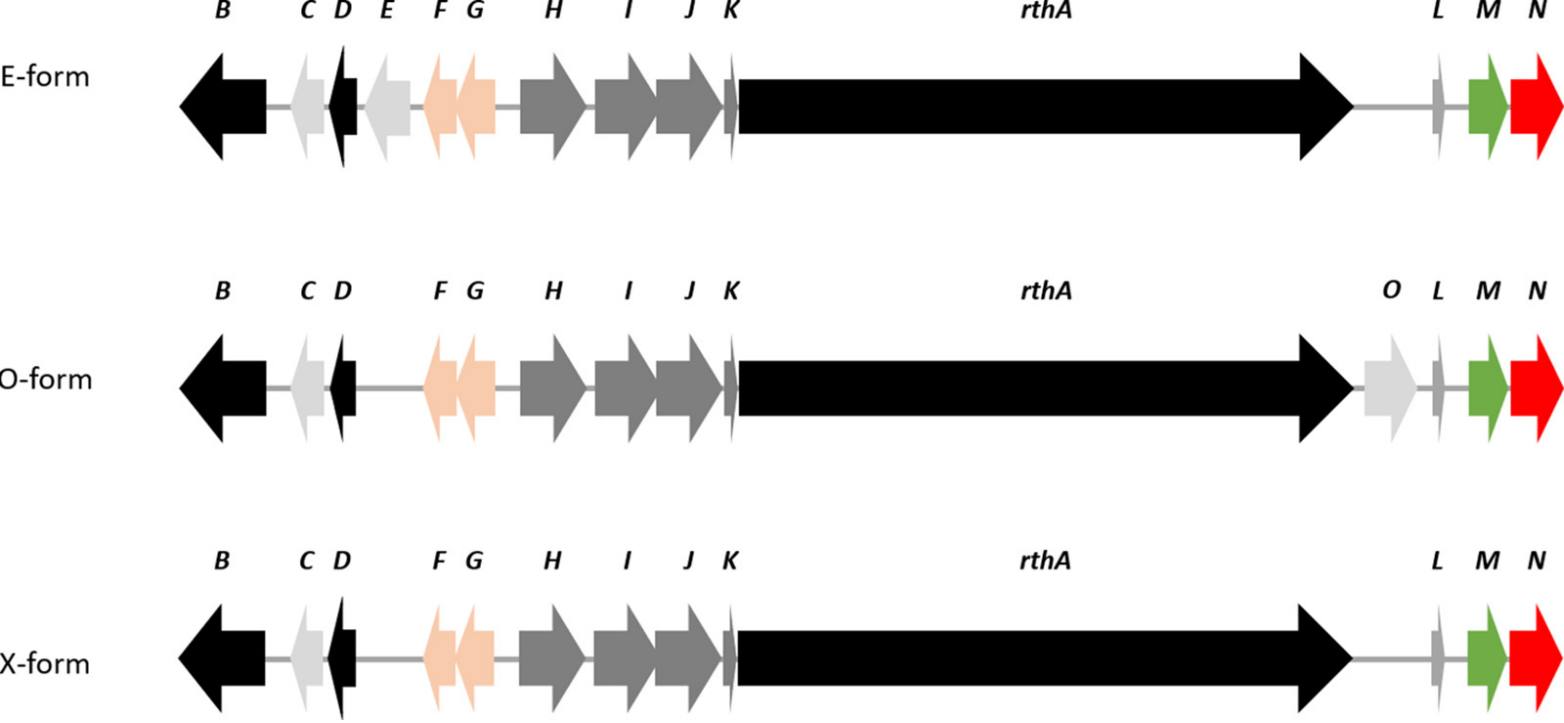
Schematic representation of E-, O-, and X-form rotihibin biosynthetic gene clusters.

**FIG 16:**
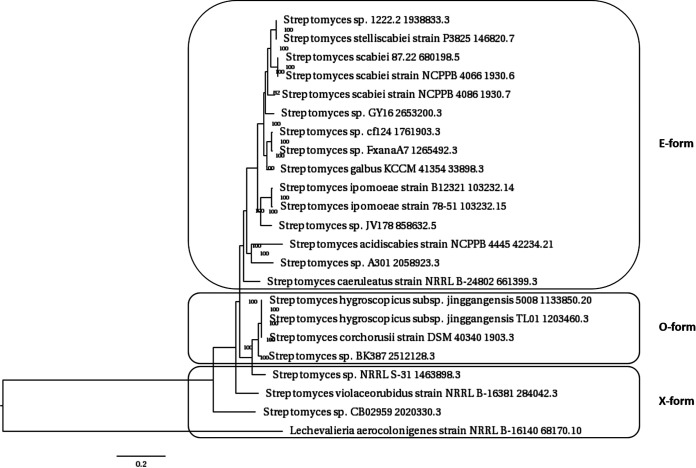
Phylogenetic tree of 23 *Actinobacteria* analyzed in this study. The phylogenetic tree was generated using the codon tree method in PATRIC.

Rotihibin BGCs exist in three different organizations (Table 3), and the gene clusters identified here were classified as E-form, O-form, and X-form clusters, based on the presence of two genes: *rthE*, encoding, a luciferease-like monogygenase (LLM) class F_420_-dependent oxidoreductase with unknown function, and *rthO*, which encodes a cytochrome P450 hydroxylase (Fig. 15). The cytochrome P450 OxyD from the vancomycin biosynthetic operon is involved in the biosynthesis of the modified amino acid β-hydroxytyrosine, while the cytochrome P450 TxtC was identified to be required for postcyclization hydroxylation of the cyclic dipeptide thaxtomin A (61, 62). The combination of this additional enzyme with additional modules in the *rthA* gene, which is discussed later, in *Actinobacteria* harboring the O-form BGC indicates the putative production of other rotihibin variants. The phylogenetic tree of the complete genomes correlates with the distribution of the different BGC forms (Fig. 16). The nucleotide sequences of the *rth* genes of the different *Actinobacteria* were compared with those of the *rth* genes of *Streptomyces scabies* 87-22 (Table 4). The presence of two rotihibin BGCs in Streptomyces rameus BK387 is quite remarkable and could be the result of recent intraspecies horizontal gene transfer (HGT) or double interspecies HGT. The loss of *rthN* in one of these BGCs could be compensated for by the other *rthN* gene.

**TABLE 4:** Nucleotide sequence comparison of *rth* genes^*a*^

Organism	% identity														
	*rthB*	*rthC*	*rthD*	*rthE*	*rthF*	*rthG*	*rthH*	*rthI*	*rthJ*	*rthK*	*rthA*	*rthO*	*rthL*	*rthM*	*rthN*
Streptomyces scabiei NCPPB 4066	98.63	99.64	99.16	99.05	99.75	99.33	99.80	98.23	99.53	99.56	99.64	X	99.52	99.40	98.98
Streptomyces galbus KCCM 41354	81.96	92.73	91.60	91.47	92.72	90.48	89.89	87.91	89.91	91.98	89.49	X	93.33	93.81	90.78
Streptomyces bottropensis cf124	80.51	93.33	92.58	91.47	91.79	90.61	88.71	85.40	90.13	92.71	89.08	X	93.81	93.51	90.31
Streptomyces bottropensis FxanaA7	80.47	93.33	92.58	91.29	91.79	90.75	88.78	85.34	90.19	92.71	88.74	X	93.33	93.61	90.31
Streptomyces ipomoeae B12321	90.65	93.45	89.08	87.73	86.83	86.86	85.60	82.34	85.39	89.82	86.69	X	93.81	94.42	92.51
Streptomyces cinereoruber subsp. *fructofermentans* GY16	81.70	92.24	88.64	89.02	89.65	85.77	88.91	84.98	87.39	91.58	86.68	X	94.76	93.00	89.76
Streptomyces ipomoeae 78-51	88.48	93.45	88.80	87.81	86.96	86.45	85.54	82.10	85.10	89.92	86.69	X	93.81	94.62	92.36
Streptomyces caeruleatus NRRL B-24802	87.05	94.06	89.08	89.57	86.38	86.86	84.99	83.23	85.18	90.53	87.35	X	94.76	93.61	88.55
Streptomyces europaeiscabiei NCPPB 4086	82.14	90.42	84.17	88.20	89.64	89.16	90.15	83.78	85.78	90.00	87.31	X	93.81	94.21	92.04
Streptomyces stelliscabiei 1222.2	80.84	92.12	86.33	91.05	89.52	89.03	87.34	86.04	86.92	90.53	87.31	X	91.22	85.68	89.20
*Streptomyces stelliscabiei* P3825	81.11	92.12	86.33	89.48	89.52	89.03	87.34	86.12	86.75	90.35	88.67	X	91.22	85.58	89.05
*Streptomyces geranii* A301	81.26	90.67	88.52	88.09	87.25	86.57	84.48	84.68	85.14	93.09	85.30	X	92.31	88.14	90.78
Streptomyces diastatochromogenes CB02959	84.58	92.12	87.54	X	87.69	86.80	84.77	84.68	86.66	85.82	86.21	X	73.27	87.61	82.58
*Streptomyces ossamyceticus* JV178	77.56	87.39	83.29	84.02	87.25	81.77	74.33	79.70	82.18	84.21	83.37	X	90.95	81.40	78.38
Streptomyces acidiscabies NCPPB 4445	73.55	85.21	80.12	81.44	81.07	79.50	73.78	76.22	78.68	88.11	80.29	X	74.63	91.71	86.77
Streptomyces rameus BK387	74.27	82.67	74.63	X	73.81	75.83	74.42	74.79	75.19	81.46	77.98	✓	81.95	76.86	75.06
Streptomyces hygroscopicus subsp. *jinggangensis* TL01	74.69	81.14	73.56	X	74.58	75.79	73.76	74.41	74.29	81.44	77.67	✓	81.46	76.37	74.55
Streptomyces jiujiangensis NRRL S-31	74.51	82.47	72.40	X	73.71	71.37	73.88	75.35	75.41	78.83	77.72	✓	81.60	77.29	76.83
Streptomyces hygroscopicus subsp. *jinggangensis* 5008	74.69	81.14	73.56	X	74.58	75.79	73.76	74.41	74.29	80.98	75.48	✓	81.46	76.37	74.55
Streptomyces corchorusii DSM 40340	74.91	81.14	73.82	X	74.58	75.79	73.82	74.32	74.45	80.49	77.64	✓	73.54	76.37	74.71
Lechevalieria aerocolonigenes NRRL B-16140	76.87	77.60	75.60	X	72.68	70.83	70.05	75.35	73.86	78.46	71.03	X	83.25	73.02	74.00
Streptomyces violaceorubidus NRRL B-16381	72.89	80.73	73.54	X	71.47	76.28	70.05	69.96	68.04	72.83	74.81	X	72.38	73.48	75.66
Streptomyces rameus BK387	71.70	79.76	72.63	X	73.37	68.18	71.18	73.02	74.02	73.08	73.39	✓	73.54	74.03	X

a The nucleotide sequences of *rth* genes of different *Actinomycetes* were compared against those of *Streptomyces scabies* 87-22 using BLAST. X: gene is missing, checkmark: gene is present.

The comparison of the *rthA* genes in the different actinomycetes displayed some NRPSs with more modules and other A-domain specificities, suggesting the production of rotihibin analogues (Table 5). These compounds are interesting and could exhibit different or better activities toward plants.

**TABLE 5:** Comparison of *rthA* genes in different *Actinomycetes*^*a*^

Strain	No. of modules	Length (aa)	Analogue						
			Module 1	Module 2	Module 3	Module 4	Module 5	Module 6	Module 7
Streptomyces violaceorubidus NRRL B-16381	4	4,235	X		NosA-M2-Ser	No hit	MycC-M2-Asx	X	X
Streptomyces caeruleatus NRRL B-24802	5	5,490	Pps4-M2-Glu		PvdD-M3-Ser	Cda2-M3-Asn	Cda2-M3-Asn	X	X
Streptomyces cinereoruber subsp. *fructofermentans* GY16	5	5,514	Pps4-M2-Glu		PvdD-M3-Ser	Cda2-M3-Asn	Cda2-M3-Asn	X	X
*Streptomyces geranii* A301	5	5,523	Pps4-M2-Glu		PvdD-M3-Ser	Cda2-M3-Asn	Cda2-M3-Asn	X	X
Streptomyces ipomoeae 78-51	5	5,458	Pps4-M2-Glu		PvdD-M3-Ser	Cda2-M3-Asn	Cda2-M3-Asn	X	X
Streptomyces ipomoeae 88-35	5	5,458	Pps4-M2-Glu		PvdD-M3-Ser	Cda2-M3-Asn	Cda2-M3-Asn	X	X
Streptomyces ipomoeae B12321	5	5,458	Pps4-M2-Glu		PvdD-M3-Ser	Cda2-M3-Asn	Cda2-M3-Asn	X	X
*Streptomyces scabies* 87-22	5	5,514	Pps4-M2-Glu		PvdD-M3-Ser	Cda2-M3-Asn	Cda2-M3-Asn	X	X
*Streptomyces scabies* NCPPB 4066	5	5,510	Pps4-M2-Glu		PvdD-M3-Ser	Cda2-M3-Asn	Cda2-M3-Asn	X	X
*Streptomyces scabies* RL-34	5	5,535	Pps4-M2-Glu		PvdD-M3-Ser	Cda2-M3-Asn	Cda2-M3-Asn	X	X
Streptomyces bottropensis cf124	5	5,517	Pps4-M2-Glu		PvdD-M3-Ser	Cda2-M3-Asn	Cda2-M3-Asn	X	X
Streptomyces bottropensis FxanaA7	5	5,517	Pps4-M2-Glu		PvdD-M3-Ser	Cda2-M3-Asn	Cda2-M3-Asn	X	X

Streptomyces acidiscabies NCPPB 4445	5	5,411	PvdD-M3-Ser		PvdD-M3-Ser	Cda2-M3-Asn	Cda2-M3-Asn	X	X

Streptomyces galbus KCCM 41354	5	5,478	Pps4-M2-Glu		PvdD-M3-Ser	No hit	Cda2-M3-Asn	X	X
Streptomyces europaeiscabiei NCPPB 4086	5	5,449	Pps4-M2-Glu		PvdD-M3-Ser	No hit	Cda2-M3-Asn	X	X

*Streptomyces stelliscabiei* 1222.2	5	5,507	Pps4-M2-Glu		Pps4-M2-Glu	Cda2-M3-Asn	Cda2-M3-Asn	X	X
*Streptomyces stelliscabiei* P3825	5	5,504	Pps4-M2-Glu		Pps4-M2-Glu	Cda2-M3-Asn	Cda2-M3-Asn	X	X

Lechevalieria aerocolonigenes NRRL B-16140	7	7,050	Pps4-M2-Glu		Pps4-M2-Glu	Cda2-M3-Asn	Cda2-M3-Asn	TycC-M2-Gln	Pps4-M2-Glu

Streptomyces diastatochromogenes CB02959	7	7,066	Pps4-M2-Glu		PvdD-M3-Ser	Cda2-M3-Asn	Cda2-M3-Asn		Pps4-M2-Glu

*Streptomyces ossamyceticus* JV178	7	7,399	Pps4-M2-Glu		PvdD-M3-Ser	Cda2-M3-Asn	MycC-M2-Asx	No hit	Pps4-M2-Glu

Streptomyces jiujiangensis S-31	7	7,036	No hit		PvdD-M3-Ser	Cda2-M3-Asn	Cda2-M3-Asn	AcmB-M2-Val	Pps4-M2-Glu

Streptomyces corchorusii DSM 40340	7	7,027	No hit		Pps4-M2-Glu	Cda2-M3-Asn	Cda2-M3-Asn	AcmB-M2-Val	Pps4-M2-Glu
Streptomyces hygroscopicus subsp. *jinggangensis* 5008	7	7,027	No hit		Pps4-M2-Glu	Cda2-M3-Asn	Cda2-M3-Asn	AcmB-M2-Val	Pps4-M2-Glu
Streptomyces hygroscopicus subsp. *jinggangensis* TL01	7	7,027	No hit		Pps4-M2-Glu	Cda2-M3-Asn	Cda2-M3-Asn	AcmB-M2-Val	Pps4-M2-Glu
Streptomyces rameus BK387	7	7,033	No hit		Pps4-M2-Glu	Cda2-M3-Asn	Cda2-M3-Asn	AcmB-M2-Val	Pps4-M2-Glu
Streptomyces rameus BK387	7	7,379	Pps4-M2-Glu		PvdD-M3-Ser	Cda2-M3-Asn	MycC-M2-Asx	AcmB-M2-Val	Pps4-M2-Glu

a The different modular organizations and A-domain specificities of the nonribosomal peptide synthase (NRPS) RthA suggest the production of rotihibin analogues (grouped together) over the investigated strains. aa, amino acids.

## MATERIALS AND METHODS

### Fermentation and analysis of *Streptomyces scabies* RL-34, 87-22, and mutant strains.

*Streptomyces scabies* RL-34, *S. scabies* 87-22, and the Δ*rthB* mutant were cultured in a shaking incubator at 250 rpm at 28°C for 4 days using borosilicate-baffled flasks with membrane screw caps containing 100 ml of ISP-4 medium, prepared as follows: 10 g soluble starch (Sigma-Aldrich), 1 g K_2_HPO_4_, 1 g MgSO_4_, 1 g NaCl, 2 g (NH_4_)_2_SO_4_, 2 g CaCO_3_, 1 mg FeSO_4_, 1 mg MnCl_2_, and 1 mg ZnSO_4_ were dissolved in 1 liter of deionized water. For the induction of rotihibin production, wild-type cultures were grown in the presence of 0.7% cellobiose (Sigma-Aldrich) or 0.5% glucose (Merck).

Cultures were centrifuged at 10,000 × *g* for 10 min, and 2 ml of each supernatant was transferred to a new tube. The samples were mixed with *n*-butanol (1:1), vortexed gently for 10 min, and centrifuged for 15 min at 16,000 × *g*. The aqueous phase of the extract was dried and stored at 20°C until further use.

The dried extract was dissolved in 10 mM ammonium formate (NH_4_FA) (pH 3) and analyzed by HPLC, monitoring the absorbance at 220 nm. The sample was eluted with solvent A (10 mM NH_4_FA, pH 3) and solvent B (acetonitrile [ACN]) using a 2-to-90% 7.5-min gradient at 1 ml/min on a Zorbax Eclipse Plus C_18_ column (4.6 by 100 mm, 3.5 μm). Fractions were collected every 15 s, corresponding to 250 μl. The total area under the curve (AUC) for each metabolite was calculated. Student’s *t* test (two tailed, homoscedastic) was executed to evaluate the differential metabolite abundances between the different conditions in a statistical way.

The fractions of interest were loaded into a Triverse Nanomate source (Advion) and, using 1.6 kV and 0.3 lb/in^2^ of pressure, electrosprayed into a Waters Synapt G1 mass spectrometer. Scans typically ranged from *m/z* 100 to 1,200, with a scan time of 1 s, for a total of 2 min. For MS/MS experiments, the collision energy was optimized to achieve good fragmentation spectra.

### Generation of the Δ*rthB* (*scab_3221*; *scab_RS01465*) deletion mutant in *Streptomyces scabies* 87-22.

Deletion of the *rthB* gene was performed in *S. scabies* 87-22 using the ReDirect PCR targeting method (63) by replacing the genomic copy of the gene with an apramycin resistance cassette: using a HindIII-EcoRI restriction fragment obtained from pIJ773 (Table 6) as the PCR template, a disruption cassette, containing the resistance gene *aac(3)IV* and *oriT* for conjugative transfer, was amplified by PCR with primers BDF33 and BDF34 and gel purified as performed previously for *cebR*, *cebE*, *msiK*, and *bglC* (11, 13, 29).

**TABLE 6:** Primers and genetic constructs used in this study^*a*^

Primer or genetic construct	Sequence (5′→3′) or description	Application, reference, or source
Primers		
BDF33 (ReDirect *SCAB_3221_f_+3*)	GGCCGTTCCGCCGCCCCGGCACTCCCACCCGGGGCGGTGATTCCGGGGATCCGTCGACC	Disruption cassette amplification
BDF34 (ReDirect *SCAB_3221_r_+2307*)	AACACCGGCCGCCGGCCCGCCCGTACCGCCGACGCTTCATGTAGGCTGGAGCTGCTTC	Disruption cassette amplification
BDF35 (*SCAB_3221_f_-682_XbaI*)	AAA*TCTAGA*GCACGAACACGAGAAGACC	PCR checking/complementation
BDF36 (*SCAB_3221_r_+2791_XbaI*)	AAA*TCTAGA*TTGGGAGACGATCTTGATGC	PCR checking/complementation
BDF69 (*SCAB_3221_fwd_+985*)	CCGCCTATGTCATCTACACC	PCR checking
BDF70 (*SCAB_3221_rev_+1097*)	CCGAAGCCGTACTCCTCG	PCR checking

Plasmids or cosmids		
pIJ773	Template for amplification of the apramycin resistance cassette [*aac(3)IV* (Apra^r^) *oriT* (RK2) FRT *amp* (Amp^r^)]	63
cos2012	Supercos-1 (Agilent) derivative containing the genomic insert of *S. scabies* 87-22 from positions 341913–386384 (Amp^r^ Kan^r^)	Isolde Francis
pIJ790	Contains the λ Red recombination under the control of an arabinose-inducible promoter (p_araBAD_) (Cml^r^)	63
cos2012Δ3221	Cosmid 2012 derivative containing the apramycin resistance cassette instead of the *rthB* gene	This study
pUZ8002	Nontransmissible plasmid supplying transfer functions for mobilization of *oriT*-containing vectors from E. coli to *Streptomyces* spp. (Kan^r^)	80
pJET1.2/blunt	E. coli plasmid used for high-efficiency blunt-end cloning of PCR products (Amp^r^)	Thermo Scientific
pBDF054	pJET1.2 derivative containing the 3,512-bp DNA fragment for *rthB* complementation	This study
pAU3-45	pSET152 derivative, integrative plasmid with a thiostrepton resistance gene inserted into the blunted NheI restriction site [*lacZ*α *ori* (pUC18) *aac(3)IV* (Apra^r^) *oriT* (RK2) *attP* (φC31) *int* (φC31) *tsr* (Thio^r^)]	64

a Engineered restriction sites are indicated in italic and underlined, non-homologous extensions are underlined.

In parallel, cosmid 2012 (cos2012), based on Supercos-1 and containing a genomic insert of *S. scabies* 87-22 ranging from positions 341913 to 386384, including *rthB*, was introduced by electroporation into Escherichia coli BW25113 carrying plasmid pIJ790 for the arabinose-inducible expression of the λ Red recombination machinery. After culture to an optical density (OD) of 0.4 with arabinose at 10 mM to induce the λ Red machinery, 50 μl of washed E. coli BW25113/pIJ790 cos2012 cells was electroporated with 100 ng (in 1 μl) of the *rthB*-targeting disruption cassette, and apramycin-resistant clones were selected on LB agar plates plus apramycin (50 μg/ml) and kanamycin (50 μg/ml). The gene replacement by the disruption cassette on the cosmid was confirmed by PCR using primers BDF35 and BDF36 (Table 6) and Sanger sequencing. The resulting cos2012Δ3221 construct was purified using a GeneJET plasmid miniprep kit (Thermo Scientific) (according to the manufacturer’s guidelines) and transferred by intergeneric conjugation with E. coli ET12567/pUZ8002 (ETpUZ) as the donor strain and *S. scabies* 87-22 as the recipient strain. After growing ETpUZ to an OD of 0.4 (50-ml culture in LB plus chloramphenicol [30 μg/ml], apramycin [50 μg/ml], kanamycin [50 μg/ml], and ampicillin [100 μg/ml]), cells were washed to remove antibiotics and mixed with *S. scabies* 87-22 spores for mating. The conjugative mixture was then plated on 30-ml petri dishes of soy flour mannitol medium (SFM) (20 g/liter mannitol, 20 g/liter soy flour, and 20 g/liter agar in tap water and autoclaved twice) plus 10 mM MgCl_2_ and overlaid with nalidixic acid (50 μg/ml) and apramycin (40 μg/ml). Exconjugants were transferred to ISP-4 agar plates plus nalidixic acid (25 μg/ml) and apramycin (50 μg/ml) to obtain uniform mutant lines that were then used to prepare spore stocks. Each *S. scabies* Δ*rthB* mutant was checked for apramycin resistance and kanamycin sensitivity on ISP-2 agar plates. Genomic DNA was extracted from 48-h liquid cultures in tryptic soy broth (TSB) using a GenElute bacterial genomic DNA kit (Sigma-Aldrich) (according to the manufacturer’s guidelines) and used as the PCR template for mutation confirmation.

### Complementation of the Δ*rthB* deletion mutant.

*rthB*, including its upstream (−701 bp) and downstream (+501 bp) regions, was amplified by PCR with primers BDF35 and BDF36 to obtain a 3,512-bp fragment flanked by two XbaI restriction sites. The PCR product was first cloned into a pJET1.2 vector, which was named pBDF054. Using the XbaI enzyme, *rthB* and surrounding regions were isolated and cloned into a pAU3-45 (64) integrative vector named pBDF043. Conjugation was performed with *S. scabies* Δ*rthB* clones 1 and 2 on SFM (plus 10 mM MgCl_2_) overlaid with apramycin (50 μg/ml), nalidixic acid (50 μg/ml), and thiostrepton (12.5 μg/ml). The integration of the plasmid was confirmed by PCR with primers BDF69 and BDF70.

### Structural confirmation by NMR spectroscopy.

Both rotihibin analogues were dissolved in 0.6 ml of DMSO-d_6_, and the nuclear magnetic resonance (NMR) spectra were recorded at 25°C on a Bruker Avance II 700-MHz spectrometer equipped with a 5-mm Prodigy TCI N_2_ cooled cryoprobe. Total correlated spectroscopy (TOCSY) spectra were recorded using the standard pulse sequences from the Bruker library.

### Protein extraction and sample preparation.

*S. scabies* RL-34 cells were cultivated in three different growth media (ISP-4 medium, ISP-4 medium supplemented with 0.7% cellobiose, and ISP-4 medium supplemented with 0.5% glucose) for 96 h (28°C at 250 rpm), with three biological replicates under each condition. The cultures were centrifuged at 10,000 × *g* for 10 min. The resulting pellet was washed two times and resuspended in 10 ml lysis buffer (1× phosphate-buffered saline [PBS], 0.1% SDS, protease inhibitor cocktail). The homogeneous mycelium suspension was sonicated (20 times with 30 s on and 30 s off at 30%) on ice using a Branson digital sonifier 450 cell disruptor. The homogenate was centrifuged at 16,000 × *g* for 30 min at 4°C. Pellets were discarded, and the supernatant was subjected to trichloroacetic acid (TCA) precipitation. The crude intracellular extract was mixed with a 100% (wt/vol) TCA solution (Sigma-Aldrich) (4:1) and incubated overnight. This mixture was centrifuged at 16,000 × *g* for 15 min at 4°C, and the protein pellet was washed two times with ice-cold acetone and solubilized in 2 M urea in 50 mM ammonium bicarbonate. The protein concentration was determined using the Pierce Coomassie protein assay kit.

Protein extracts (10 μg) were spiked with bovine serum albumin (BSA) (MS-grade protein standard) (0.2 ng/μl) as an internal standard. The proteins were subsequently reduced with 2.5 mM dithiothreitol (DTT) (10 min at 60°C), alkylated with 7.5 mM iodoacetamide (20 min at room temperature), and trypsinized (1/50) (overnight at 37°C). The reaction was stopped with 1% HCOOH. After centrifugation (30 min at 13,200 rpm), the samples were filtered with a prewashed 0.22-μm Costar filter and transferred to clean tubes.

### Targeted proteomic analysis.

The resulting peptide solutions were injected (0.1 μg) onto an ultraperformance liquid chromatography (UPLC) M-Class system where the peptides were trapped for 5 min, at 15 μl/min, on a 300-μm by 50-mm, 5-μm, 100-Å Acquity UPLC M-Class symmetry C_18_ trap column (Waters) and separated on the iKey separation device (150 μm by 100 mm, 1.8 μm) (HSS T3; Waters) with solvent A (0.1% HCOOH in H_2_O [Biosolve]) and solvent B (0.1% HCOOH in ACN [Biosolve]) using a 20-min gradient of 3 to 50% solvent B at a flow rate of 1 μl/min. The strong and weak solutions used to wash the autosampler were 0.1% HCOOH in H_2_O and 0.1% HCOOH in acetonitrile-water-isopropanol (50:25:25, vol/vol/vol), respectively. The separated peptides were introduced into the IonKey source coupled to a Waters Xevo TQ-S triple-quadrupole mass spectrometer for quantification of the analytes in the positive-ion mode (ESI^+^). The ESI-MS/MS parameters were as follows: capillary voltage of 3.6 kV, cone voltage of 40 V, source temperature of 120°C, and collision gas argon flow rate of 0.19 ml/min. The transitions of the selected precursor ions were detected in MRM mode at different collision energies specific for each precursor (Table 7). Data were acquired using MassLynx 4.1 software and processed in Skyline 3.7. The transition curves were subjected to Savitsky-Golay smoothing, and their area under the curve (AUC) was determined and normalized to the spiked BSA standard. Student’s *t* test (two tailed, homoscedastic) was executed to evaluate the differential protein abundances between the different conditions in a statistical way.

**TABLE 7:** Transitions selected for MRM validation of Rth proteins upon cellobiose and glucose supply^*a*^

Peptide	Precursor ion mass (m/z)	CE (V)	Fragment ion mass (m/z)
RthA			
AWIDSDLATPVPVTGER	913.968^++^	33	L [y11]—1,139.642^+^
			A [y10]—1,026.558^+^
			T [y9]—955.521^+^
ADTSGDPTFEELLDR	833.384^++^	30	P [y9]—1,119.568^+^
			T [y8]—1,022.515^+^
			F [y7]—921.468^+^
GGTVPFAVPAALR	628.362^++^	22	A [y9]—941.557^+^
			V [y8]—844.504^+^
			P [y7]—697.436^+^

RthH			
VTDEQLAALDLSR	715.878^++^	25	Q [y9]—986.563^+^
			L [y8]—858.504^+^
			A [y7]—745.420^+^
EDPLLTDALAGQR	699.865^++^	25	L [y9]—944.516^+^
			T [y8]—831.432^+^
			D [y7]—730.384^+^

RthD			
LIDEEPYR	517.761^++^	18	D [y6]—808.347^+^
			E [y5]—693.320^+^
			E [y4]—564.278^+^
ATGLSDEEFLAR	654.825^++^	23	S [y8]—966.453^+^
			D [y7]—879.421^+^
			E [y6]—764.394^+^

RthE			
IPVYLAALGPK	571.353^++^	20	V [y9]—931.561^+^
			Y [y8]—832.493^+^
			L [y7]—669.430^+^
IDVGSAVLQIPAR	669.891^++^	24	A [y8]—867.541^+^
			V [y7]—796.504^+^
			L [y6]—697.436^+^

RthI			
GQLPEGAWR	507.262^++^	18	L [y7]—828.436^+^
			P [y6]—715.352^+^
			E [y5]—618.299^+^
LGTADLWLR	522.795^++^	18	A [y6]—773.430^+^
			D [y5]—702.393^+^
			L [y4]—587.366^+^

RthJ			
LYGGAATDIPHVR	685.365^++^	24	A [y8]—908.495^+^
			T [y7]—837.458^+^
			D [y6]—736.410^+^
SELAGVFADLLR	645.856^++^	23	G [y8]—890.509^+^
			V [y7]—833.488^+^
			F [y6]—734.420^+^

a CE, collision energy.

### Bioinformatics analysis of the rotihibin biosynthetic gene cluster.

Prediction of the gene cluster of *Streptomyces scabies* 87-22 was performed using antiSMASH 3.0 (30). The sequence of the biosynthetic rotihibin gene cluster was further analyzed and annotated using UniProt and BLAST (65, 66). Similar gene clusters were identified in other *Actinomycetes* strains by PATRIC 3.5.21 analysis (31). A phylogenetic tree was calculated, from the *rthA* MUSCLE sequence alignment, using the neighbor-joining method with MEGA-X (67). Thaxtomin synthetase A (*txtA*) was selected as the outgroup.

The module/domain organization of the different NRPSs in the rotihibin gene cluster was predicted via PRISM (68, 69). The A-domain specificity was predicted via four different software tools: the LSI-based A-domain function predictor, NRPSsp, the PKS/NRPS Web server, and SEQL-NRPS. The LSI-based predictor uses latent semantic indexing to predict adenylation domain specificities (70). NRPSsp uses hits against hidden Markov model (HMM) databases to predict specificities of NRPS adenylation domains (71). The PKS/NRPS Web server uses BLAST to detect catalytic domains in NRPS and predicts A-domain specificities by comparing signatures of A domains with those of known substrates (72). Finally, SEQL-NRPS predicts A-domain specificities using the discriminative classification method sequence learner (SEQL) (73).

### *Lemna minor* L. and Arabidopsis thaliana L. Heynh. bioassay.

A sterile 24-well plate was used to grow *Lemna minor* (duckweed) in 2 ml mineral medium [11.1 mg CaCl_2_, 202 mg KNO_3_, 49.6 mg MgSO_4_·7H_2_O, 50.3 mg KH_2_PO_4_, 27.8 mg K_2_HPO_4_, 6 mg FeSO_4_·7H_2_O, 17.4 mg K_2_SO_4_, 5.72 mg H_3_BO_3_, 2.82 mg MnCl_2_·4H_2_O, 0.6 mg ZnSO_4_, 10 mg Na_2_-EDTA, 0.008 mg CuCl_2_·H_2_O, 0.054 mg CoCl_2_·6H_2_O, and 0.043 mg (NH_4_)_6_Mo_7_O_24_ dissolved in 1 liter of MilliQ (MQ) water (pH 6.5 ± 0.1)] (74). The plants were incubated in quadruple for 4 days in a growth chamber (16-h light exposure at 22°C). In order to assess the sensitivity of *L. minor* to rotihibins C and D, a wide concentration range (0.17 to 84.4 μM for rotihibin C and 0.3 to 157.6 μM for rotihibin D) was tested. After 4 days of incubation, the plates were analyzed based on the growth and maximal photochemistry efficiency of photosystem II via chlorophyll fluorescence (*F*_V_/*F*_M_) of the duckweed plants.

Arabidopsis thaliana ecotype Col-0 pregerminated seeds were transferred to petri dishes with half-strength Murashige-Skoog (MS) medium without sucrose (0.22% MS salts and 0.8% plant agar) (75). Nine days after germination, the plants were treated with rotihibins C and D dissolved in water (0.005 to 0.1 mM) via nebulization on the leaves. After 4 days of treatment, the plates were analyzed based on the growth and *F*_V_/*F*_M_ values of the *Arabidopsis* plants.

Imaging was achieved with an in-house-developed phenotyping platform, in an environment-controlled growth chamber, property of the Ghent University Laboratory of Applied Mycology and Phenomics. The platform allows visualization of diverse physiological traits via a multispectral 3CCD camera equipped with 12 interference filters in real time, based on specific absorption, reflection, and emission patterns, such as leaf surface, efficiency of photosynthesis, chlorophyll and anthocyanin contents, and green fluorescent protein (GFP)-tagged organisms. This platform is equipped with a dispenser, which can be fitted with a nozzle to treat the plants with rotihibins or other agrochemicals in a standardized manner. Image data processing was performed using Data Analysis (version 5.4.6; Phenovation, Wageningen, Netherlands), and statistical analysis was performed in RStudio (version 1.1.383) (76) using R (version 4.0.5) (77) for Welch’s *t* test and Tukey’s *post hoc* test. Visualization of the data was done using the ggplot2 package (78).

### Data availability.

The MRM data have been deposited in PeptideAtlas (79) with the data set identifier PASS01674.

## Supplementary Material

Reviewer comments

## References

[1] Loria R, Kers J, Joshi M. 2006. Evolution of plant pathogenicity in *Streptomyces*. Annu Rev Phytopathol 44:469–487. doi:10.1146/annurev.phyto.44.032905.091147. 16719719

[2] Lerat S, Simao-Beaunoir AM, Beaulieu C. 2009. Genetic and physiological determinants of *Streptomyces scabies* pathogenicity. Mol Plant Pathol 10:579–585. doi:10.1111/j.1364-3703.2009.00561.x. 19694949 PMC6640508

[3] Bouchek-Mechiche K, Gardan L, Normand P, Jouan B. 2000. DNA relatedness among strains of Streptomyces pathogenic to potato in France: description of three new species, S. europaeiscabiei sp. nov. and S. stelliscabiei sp. nov. associated with common scab, and S. reticuliscabiei sp. nov. associated with netted scab. Int J Syst Evol Microbiol 50(Part 1):91–99. doi:10.1099/00207713-50-1-91. 10826791

[4] Faucher E, Otrysko B, Paradis É, Hodge NC, Stall RE, Beaulieu C. 1993. Characterization of streptomycetes causing russet scab in Québec. Plant Dis 77:1217–1220. doi:10.1094/PD-77-1217.

[5] Guan D, Grau BL, Clark CA, Taylor CM, Loria R, Pettis GS. 2012. Evidence that thaxtomin C is a pathogenicity determinant of *Streptomyces ipomoeae*, the causative agent of Streptomyces soil rot disease of sweet potato. Mol Plant Microbe Interact 25:393–401. doi:10.1094/MPMI-03-11-0073. 22088193

[6] King RR, Lawrence CH, Clark MC, Calhoun LA. 1989. Isolation and characterization of phytotoxins associated with *Streptomyces scabies*. J Chem Soc Chem Commun (Camb) 1989:849–850. doi:10.1039/c39890000849.

[7] Loria R, Bignell DR, Moll S, Huguet-Tapia JC, Joshi MV, Johnson EG, Seipke RF, Gibson DM. 2008. Thaxtomin biosynthesis: the path to plant pathogenicity in the genus *Streptomyces*. Antonie Van Leeuwenhoek 94:3–10. doi:10.1007/s10482-008-9240-4. 18392685

[8] Bischoff V, Cookson SJ, Wu S, Scheible WR. 2009. Thaxtomin A affects CESA-complex density, expression of cell wall genes, cell wall composition, and causes ectopic lignification in *Arabidopsis thaliana* seedlings. J Exp Bot 60:955–965. doi:10.1093/jxb/ern344. 19269997 PMC2652064

[9] Scheible WR, Fry B, Kochevenko A, Schindelasch D, Zimmerli L, Somerville S, Loria R, Somerville CR. 2003. An Arabidopsis mutant resistant to thaxtomin A, a cellulose synthesis inhibitor from *Streptomyces* species. Plant Cell 15:1781–1794. doi:10.1105/tpc.013342. 12897252 PMC167169

[10] Wach MJ, Krasnoff SB, Loria R, Gibson DM. 2007. Effect of carbohydrates on the production of thaxtomin A by *Streptomyces acidiscabies*. Arch Microbiol 188:81–88. doi:10.1007/s00203-007-0225-x. 17340119

[11] Jourdan S, Francis IM, Kim MJ, Salazar JJ, Planckaert S, Frere JM, Matagne A, Kerff F, Devreese B, Loria R, Rigali S. 2016. The CebE/MsiK transporter is a doorway to the cello-oligosaccharide-mediated induction of *Streptomyces scabies* pathogenicity. Sci Rep 6:27144. doi:10.1038/srep27144. 27250236 PMC4890002

[12] Joshi MV, Bignell DR, Johnson EG, Sparks JP, Gibson DM, Loria R. 2007. The AraC/XylS regulator TxtR modulates thaxtomin biosynthesis and virulence in *Streptomyces scabies*. Mol Microbiol 66:633–642. doi:10.1111/j.1365-2958.2007.05942.x. 17919290

[13] Francis IM, Jourdan S, Fanara S, Loria R, Rigali S. 2015. The cellobiose sensor CebR is the gatekeeper of Streptomyces scabies pathogenicity. mBio 6:e02018-14. doi:10.1128/mBio.02018-14. 25714708 PMC4358012

[14] Bignell DR, Francis IM, Fyans JK, Loria R. 2014. Thaxtomin A production and virulence are controlled by several *bld* gene global regulators in *Streptomyces scabies*. Mol Plant Microbe Interact 27:875–885. doi:10.1094/MPMI-02-14-0037-R. 24678834

[15] Planckaert S, Jourdan S, Francis IM, Deflandre B, Rigali S, Devreese B. 2018. Proteomic response to thaxtomin phytotoxin elicitor cellobiose and to deletion of cellulose utilization regulator CebR in *Streptomyces scabies*. J Proteome Res 17:3837–3852. doi:10.1021/acs.jproteome.8b00528. 30229651

[16] Natsume M, Ryu R, Abe H. 1996. Production of phytotoxins, concanamycins A and B by *Streptomyces* spp. causing potato [Solanum tuberosum] scab. Ann Phytopathol Soc Jpn 62:411–413. doi:10.3186/jjphytopath.62.411.

[17] Natsume M, Tashiro N, Doi A, Nishi Y, Kawaide H. 2017. Effects of concanamycins produced by *Streptomyces scabies* on lesion type of common scab of potato. J Gen Plant Pathol 83:78–82. doi:10.1007/s10327-017-0696-9.

[18] Bignell DR, Seipke RF, Huguet-Tapia JC, Chambers AH, Parry RJ, Loria R. 2010. *Streptomyces scabies* 87-22 contains a coronafacic acid-like biosynthetic cluster that contributes to plant-microbe interactions. Mol Plant Microbe Interact 23:161–175. doi:10.1094/MPMI-23-2-0161. 20064060

[19] Natsume M, Komiya M, Koyanagi F, Tashiro N, Kawaide H, Abe H. 2005. Phytotoxin produced by *Streptomyces* sp. causing potato russet scab in Japan. J Gen Plant Pathol 71:364–369. doi:10.1007/s10327-005-0211-6.

[20] Cao Z, Khodakaramian G, Arakawa K, Kinashi H. 2012. Isolation of borrelidin as a phytotoxic compound from a potato pathogenic *streptomyces* strain. Biosci Biotechnol Biochem 76:353–357. doi:10.1271/bbb.110799. 22313786

[21] Park DH, Kim JS, Kwon SW, Wilson C, Yu YM, Hur JH, Lim CK. 2003. *Streptomyces luridiscabiei* sp. nov., *Streptomyces puniciscabiei* sp. nov. and *Streptomyces niveiscabiei* sp. nov., which cause potato common scab disease in Korea. Int J Syst Evol Microbiol 53:2049–2054. doi:10.1099/ijs.0.02629-0. 14657144

[22] Natsume M, Nagagata A, Aittamaa M, Okaniwa N, Somervuo P, Fiedler H-P, Kreuze JF, Rokka V-M, Bång H, Kawaide H, Valkonen JPT. 2018. Phytotoxin produced by the netted scab pathogen, *Streptomyces turgidiscabies* strain 65, isolated in Sweden. J Gen Plant Pathol 84:108–117. doi:10.1007/s10327-018-0765-8.

[23] Fukuchi N, Furihata K, Takayama S, Isogai A, Suzuki A. 1992. Rotihibin A, a novel plant growth regulator, from *Streptomyces* sp. Biosci Biotechnol Biochem 56:840–841. doi:10.1271/bbb.56.840. 1368346

[24] Fukuchi N, Nakayama J, Takayama S, Isogai A, Suzuki A. 1992. Structural elucidation of rotihibin B by tandem mass spectrometry. Biosci Biotechnol Biochem 56:1152–1153. doi:10.1271/bbb.56.1152. 27286399

[25] Fukuchi N, Furihata K, Nakayama J, Goudo T, Takayama S, Isogai A, Suzuki A. 1995. Rotihibins, novel plant growth regulators from *Streptomyces graminofaciens*. J Antibiot (Tokyo) 48:1004–1010. doi:10.7164/antibiotics.48.1004. 7592044

[26] Halder V, Oeljeklaus J, Heilmann G, Krahn JH, Liu Y, Xiong Y, Schlicht M, Schillinger J, Kracher B, Ehrmann M, Kombrink E, Kaschani F, Kaiser M. 2018. Identification of the natural product rotihibin A as a TOR kinase signaling inhibitor by unbiased transcriptional profiling. Chemistry 24:12500–12504. doi:10.1002/chem.201802647. 29932252

[27] Mohimani H, Gurevich A, Mikheenko A, Garg N, Nothias LF, Ninomiya A, Takada K, Dorrestein PC, Pevzner PA. 2017. Dereplication of peptidic natural products through database search of mass spectra. Nat Chem Biol 13:30–37. doi:10.1038/nchembio.2219. 27820803 PMC5409158

[28] el-Sayed ES. 2000. Production of thaxtomin A by two species of *Streptomyces* causing potato scab. Folia Microbiol (Praha) 45:415–422. doi:10.1007/BF02817614. 11357862

[29] Jourdan S, Francis IM, Deflandre B, Tenconi E, Riley J, Planckaert S, Tocquin P, Martinet L, Devreese B, Loria R, Rigali S. 2018. Contribution of the beta-glucosidase BglC to the onset of the pathogenic lifestyle of *Streptomyces scabies*. Mol Plant Pathol 19:1480–1490. doi:10.1111/mpp.12631. 29077242 PMC6638027

[30] Weber T, Blin K, Duddela S, Krug D, Kim HU, Bruccoleri R, Lee SY, Fischbach MA, Muller R, Wohlleben W, Breitling R, Takano E, Medema MH. 2015. antiSMASH 3.0—a comprehensive resource for the genome mining of biosynthetic gene clusters. Nucleic Acids Res 43:W237–W243. doi:10.1093/nar/gkv437. 25948579 PMC4489286

[31] Wattam AR, Brettin T, Davis JJ, Gerdes S, Kenyon R, Machi D, Mao C, Olson R, Overbeek R, Pusch GD, Shukla MP, Stevens R, Vonstein V, Warren A, Xia F, Yoo H. 2018. Assembly, annotation, and comparative genomics in PATRIC, the All Bacterial Bioinformatics Resource Center. Methods Mol Biol 1704:79–101. doi:10.1007/978-1-4939-7463-4_4. 29277864

[32] Yin X, Zabriskie TM. 2006. The enduracidin biosynthetic gene cluster from *Streptomyces fungicidicus*. Microbiology (Reading) 152:2969–2983. doi:10.1099/mic.0.29043-0. 17005978

[33] Kim M-S, Bae M, Jung Y-E, Kim JM, Hwang S, Song MC, Ban YH, Bae ES, Hong S, Lee SK, Cha S-S, Oh D-C, Yoon YJ. 7 May 2021. Unprecedented noncanonical features of the nonlinear nonribosomal peptide synthetase assembly line for WS9326A biosynthesis. Angew Chem Int Ed Engl. doi:10.1002/anie.202103872. 33963654

[34] Manavalan B, Murugapiran SK, Lee G, Choi S. 2010. Molecular modeling of the reductase domain to elucidate the reaction mechanism of reduction of peptidyl thioester into its corresponding alcohol in non-ribosomal peptide synthetases. BMC Struct Biol 10:1. doi:10.1186/1472-6807-10-1. 20067617 PMC2835699

[35] Thomas MG, Chan YA, Ozanick SG. 2003. Deciphering tuberactinomycin biosynthesis: isolation, sequencing, and annotation of the viomycin biosynthetic gene cluster. Antimicrob Agents Chemother 47:2823–2830. doi:10.1128/AAC.47.9.2823-2830.2003. 12936980 PMC182626

[36] Felnagle EA, Rondon MR, Berti AD, Crosby HA, Thomas MG. 2007. Identification of the biosynthetic gene cluster and an additional gene for resistance to the antituberculosis drug capreomycin. Appl Environ Microbiol 73:4162–4170. doi:10.1128/AEM.00485-07. 17496129 PMC1932801

[37] Drake EJ, Cao J, Qu J, Shah MB, Straubinger RM, Gulick AM. 2007. The 1.8 A crystal structure of PA2412, an MbtH-like protein from the pyoverdine cluster of Pseudomonas aeruginosa. J Biol Chem 282:20425–20434. doi:10.1074/jbc.M611833200. 17502378

[38] Lee KS, Lee BM, Ryu JH, Kim DH, Kim YH, Lim SK. 2016. Increased vancomycin production by overexpression of MbtH-like protein in *Amycolatopsis orientalis* KFCC10990P. Lett Appl Microbiol 63:222–228. doi:10.1111/lam.12617. 27432613

[39] Lautru S, Oves-Costales D, Pernodet JL, Challis GL. 2007. MbtH-like protein-mediated cross-talk between non-ribosomal peptide antibiotic and siderophore biosynthetic pathways in *Streptomyces coelicolor* M145. Microbiology (Reading) 153:1405–1412. doi:10.1099/mic.0.2006/003145-0. 17464054

[40] Li Y, Liu J, Adekunle D, Bown L, Tahlan K, Bignell DRD. 2019. TxtH is a key component of the thaxtomin biosynthetic machinery in the potato common scab pathogen *Streptomyces scabies*. Mol Plant Pathol 20:1379–1393. doi:10.1111/mpp.12843. 31282068 PMC6792134

[41] Neary JM, Powell A, Gordon L, Milne C, Flett F, Wilkinson B, Smith CP, Micklefield J. 2007. An asparagine oxygenase (AsnO) and a 3-hydroxyasparaginyl phosphotransferase (HasP) are involved in the biosynthesis of calcium-dependent lipopeptide antibiotics. Microbiology (Reading) 153:768–776. doi:10.1099/mic.0.2006/002725-0. 17322197

[42] Miao V, Brost R, Chapple J, She K, Gal MF, Baltz RH. 2006. The lipopeptide antibiotic A54145 biosynthetic gene cluster from *Streptomyces fradiae*. J Ind Microbiol Biotechnol 33:129–140. doi:10.1007/s10295-005-0028-5. 16208464

[43] Saum SH, Muller V. 2008. Growth phase-dependent switch in osmolyte strategy in a moderate halophile: ectoine is a minor osmolyte but major stationary phase solute in *Halobacillus halophilus*. Environ Microbiol 10:716–726. doi:10.1111/j.1462-2920.2007.01494.x. 18093162

[44] Vandenende CS, Vlasschaert M, Seah SY. 2004. Functional characterization of an aminotransferase required for pyoverdine siderophore biosynthesis in *Pseudomonas aeruginosa* PAO1. J Bacteriol 186:5596–5602. doi:10.1128/JB.186.17.5596-5602.2004. 15317763 PMC516838

[45] Hojati Z, Milne C, Harvey B, Gordon L, Borg M, Flett F, Wilkinson B, Sidebottom PJ, Rudd BA, Hayes MA, Smith CP, Micklefield J. 2002. Structure, biosynthetic origin, and engineered biosynthesis of calcium-dependent antibiotics from *Streptomyces coelicolor*. Chem Biol 9:1175–1187. doi:10.1016/s1074-5521(02)00252-1. 12445768

[46] Miao V, Coeffet-Legal MF, Brian P, Brost R, Penn J, Whiting A, Martin S, Ford R, Parr I, Bouchard M, Silva CJ, Wrigley SK, Baltz RH. 2005. Daptomycin biosynthesis in *Streptomyces roseosporus*: cloning and analysis of the gene cluster and revision of peptide stereochemistry. Microbiology (Reading) 151:1507–1523. doi:10.1099/mic.0.27757-0. 15870461

[47] Galica T, Hrouzek P, Mares J. 2017. Genome mining reveals high incidence of putative lipopeptide biosynthesis NRPS/PKS clusters containing fatty acyl-AMP ligase genes in biofilm-forming cyanobacteria. J Phycol 53:985–998. doi:10.1111/jpy.12555. 28632895

[48] Hoertz AJ, Hamburger JB, Gooden DM, Bednar MM, McCafferty DG. 2012. Studies on the biosynthesis of the lipodepsipeptide antibiotic ramoplanin A2. Bioorg Med Chem 20:859–865. doi:10.1016/j.bmc.2011.11.062. 22222159

[49] Traxler MF, Watrous JD, Alexandrov T, Dorrestein PC, Kolter R. 2013. Interspecies interactions stimulate diversification of the *Streptomyces coelicolor* secreted metabolome. mBio 4:e00459-13. doi:10.1128/mBio.00459-13. 23963177 PMC3747584

[50] Li W, Sharma M, Kaur P. 2014. The DrrAB efflux system of *Streptomyces peucetius* is a multidrug transporter of broad substrate specificity. J Biol Chem 289:12633–12646. doi:10.1074/jbc.M113.536136. 24634217 PMC4007453

[51] Bown L, Altowairish MS, Fyans JK, Bignell DR. 2016. Production of the *Streptomyces scabies* coronafacoyl phytotoxins involves a novel biosynthetic pathway with an F420-dependent oxidoreductase and a short-chain dehydrogenase/reductase. Mol Microbiol 101:122–135. doi:10.1111/mmi.13378. 26991928

[52] Baker NR. 2008. Chlorophyll fluorescence: a probe of photosynthesis in vivo. Annu Rev Plant Biol 59:89–113. doi:10.1146/annurev.arplant.59.032607.092759. 18444897

[53] Li X, Cai W, Liu Y, Li H, Fu L, Liu Z, Xu L, Liu H, Xu T, Xiong Y. 2017. Differential TOR activation and cell proliferation in *Arabidopsis* root and shoot apexes. Proc Natl Acad Sci U S A 114:2765–2770. doi:10.1073/pnas.1618782114. 28223530 PMC5347562

[54] Shimizu M, Meguro A, Hasegawa S, Nishimura T, Kunoh H. 2006. Disease resistance induced by nonantagonistic endophytic *Streptomyces* spp. on tissue-cultured seedlings of rhododendron. J Gen Plant Pathol 72:351–354. doi:10.1007/s10327-006-0305-9.

[55] Mingma R, Pathom-Aree W, Trakulnaleamsai S, Thamchaipenet A, Duangmal K. 2014. Isolation of rhizospheric and roots endophytic actinomycetes from *Leguminosae* plant and their activities to inhibit soybean pathogen, *Xanthomonas campestris* pv. *glycine*. World J Microbiol Biotechnol 30:271–280. doi:10.1007/s11274-013-1451-9. 23913026

[56] Li X, Lai X, Gan L, Long X, Hou Y, Zhang Y, Tian Y. 2018. *Streptomyces geranii* sp. nov., a novel endophytic actinobacterium isolated from root of *Geranium carolinianum* L. Int J Syst Evol Microbiol 68:2562–2567. doi:10.1099/ijsem.0.002876. 29944094

[57] Yu Y, Bai L, Minagawa K, Jian X, Li L, Li J, Chen S, Cao E, Mahmud T, Floss HG, Zhou X, Deng Z. 2005. Gene cluster responsible for validamycin biosynthesis in *Streptomyces hygroscopicus* subsp. *jinggangensis* 5008. Appl Environ Microbiol 71:5066–5076. doi:10.1128/AEM.71.9.5066-5076.2005. 16151088 PMC1214664

[58] Franco CM, Borde UP, Vijayakumar EK, Chatterjee S, Blumbach J, Ganguli BN. 1991. Butalactin, a new butanolide antibiotic. Taxonomy, fermentation, isolation and biological activity. J Antibiot (Tokyo) 44:225–231. doi:10.7164/antibiotics.44.225. 2010359

[59] Tamreihao K, Ningthoujam DS, Nimaichand S, Singh ES, Reena P, Singh SH, Nongthomba U. 2016. Biocontrol and plant growth promoting activities of a *Streptomyces corchorusii* strain UCR3-16 and preparation of powder formulation for application as biofertilizer agents for rice plant. Microbiol Res 192:260–270. doi:10.1016/j.micres.2016.08.005. 27664745

[60] Nettleton DE, Doyle TW, Krishnan B, Matsumoto GK, Clardy J. 1985. Isolation and structure of rebeccamycin—a new antitumor antibiotic from Nocardia aerocoligenes. Tetrahedron Lett 26:4011–4014. doi:10.1016/S0040-4039(00)89280-1.

[61] Cryle MJ, Meinhart A, Schlichting I. 2010. Structural characterization of OxyD, a cytochrome P450 involved in beta-hydroxytyrosine formation in vancomycin biosynthesis. J Biol Chem 285:24562–24574. doi:10.1074/jbc.M110.131904. 20519494 PMC2915692

[62] Healy FG, Krasnoff SB, Wach M, Gibson DM, Loria R. 2002. Involvement of a cytochrome P450 monooxygenase in thaxtomin A biosynthesis by *Streptomyces acidiscabies*. J Bacteriol 184:2019–2029. doi:10.1128/JB.184.7.2019-2029.2002. 11889110 PMC134914

[63] Gust B, Challis GL, Fowler K, Kieser T, Chater KF. 2003. PCR-targeted *Streptomyces* gene replacement identifies a protein domain needed for biosynthesis of the sesquiterpene soil odor geosmin. Proc Natl Acad Sci U S A 100:1541–1546. doi:10.1073/pnas.0337542100. 12563033 PMC149868

[64] Bignell DR, Tahlan K, Colvin KR, Jensen SE, Leskiw BK. 2005. Expression of ccaR, encoding the positive activator of cephamycin C and clavulanic acid production in *Streptomyces clavuligerus*, is dependent on bldG. Antimicrob Agents Chemother 49:1529–1541. doi:10.1128/AAC.49.4.1529-1541.2005. 15793135 PMC1068620

[65] Altschul SF, Gish W, Miller W, Myers EW, Lipman DJ. 1990. Basic local alignment search tool. J Mol Biol 215:403–410. doi:10.1016/S0022-2836(05)80360-2. 2231712

[66] UniProt Consortium. 2018. UniProt: the universal protein knowledgebase. Nucleic Acids Res 46:2699. doi:10.1093/nar/gky092. 29425356 PMC5861450

[67] Kumar S, Stecher G, Tamura K. 2016. MEGA7: Molecular Evolutionary Genetics Analysis version 7.0 for bigger datasets. Mol Biol Evol 33:1870–1874. doi:10.1093/molbev/msw054. 27004904 PMC8210823

[68] Skinnider MA, Dejong CA, Rees PN, Johnston CW, Li H, Webster AL, Wyatt MA, Magarvey NA. 2015. Genomes to natural products PRediction Informatics for Secondary Metabolomes (PRISM). Nucleic Acids Res 43:9645–9662. doi:10.1093/nar/gkv1012. 26442528 PMC4787774

[69] Skinnider MA, Johnston CW, Edgar RE, Dejong CA, Merwin NJ, Rees PN, Magarvey NA. 2016. Genomic charting of ribosomally synthesized natural product chemical space facilitates targeted mining. Proc Natl Acad Sci U S A 113:E6343–E6351. doi:10.1073/pnas.1609014113. 27698135 PMC5081660

[70] Baranasic D, Zucko J, Diminic J, Gacesa R, Long PF, Cullum J, Hranueli D, Starcevic A. 2014. Predicting substrate specificity of adenylation domains of nonribosomal peptide synthetases and other protein properties by latent semantic indexing. J Ind Microbiol Biotechnol 41:461–467. doi:10.1007/s10295-013-1322-2. 24104398

[71] Prieto C. 2016. Characterization of nonribosomal peptide synthetases with NRPSsp. Methods Mol Biol 1401:273–278. doi:10.1007/978-1-4939-3375-4_17. 26831714

[72] Bachmann BO, Ravel J. 2009. Chapter 8. Methods for *in silico* prediction of microbial polyketide and nonribosomal peptide biosynthetic pathways from DNA sequence data. Methods Enzymol 458:181–217. doi:10.1016/S0076-6879(09)04808-3. 19374984

[73] Knudsen M, Sondergaard D, Tofting-Olesen C, Hansen FT, Brodersen DE, Pedersen CN. 2016. Computational discovery of specificity-conferring sites in non-ribosomal peptide synthetases. Bioinformatics 32:325–329. doi:10.1093/bioinformatics/btv600. 26471456

[74] Megateli S, Dosnon-Olette R, Trotel-Aziz P, Geffard A, Semsari S, Couderchet M. 2013. Simultaneous effects of two fungicides (copper and dimethomorph) on their phytoremediation using *Lemna minor*. Ecotoxicology 22:683–692. doi:10.1007/s10646-013-1060-2. 23504441

[75] Murashige T, Skoog F. 1962. A revised medium for rapid growth and bio assays with tobacco tissue cultures. Physiol Plant 15:473–497. doi:10.1111/j.1399-3054.1962.tb08052.x.

[76] RStudio Team. 2020. RStudio: integrated development for R. RStudio, PBC, Boston, MA.

[77] R Core Team. 2013. R: a language and environment for statistical computing. R Foundation for Statistical Computing, Vienna, Austria.

[78] Wickham H. 2011. ggplot2. WIREs Comp Stat 3:180–185. doi:10.1002/wics.147.

[79] Desiere F, Deutsch EW, King NL, Nesvizhskii AI, Mallick P, Eng J, Chen S, Eddes J, Loevenich SN, Aebersold R. 2006. The PeptideAtlas project. Nucleic Acids Res 34:D655–D658. doi:10.1093/nar/gkj040. 16381952 PMC1347403

[80] Kieser T, Bibb MJ, Buttner MJ, Chater KF, Hopwood DA. 2000. Practical Streptomyces genetics. John Innes Foundation, Norwich, United Kingdom.

