# Supplementary material for: Identification of Novel Rotihibin Analogues in Streptomyces scabies, Including Discovery of Its Biosynthetic Gene Cluster
Source: Microbiol Spectr. 2021 Aug 4;9(1):10.1128/spectrum.00571-21. doi: 10.1128/spectrum.00571-21 (PMC8552735; doi:10.1128/spectrum.00571-21)
Supplement: SUPPLEMENTAL FILE 1 — Supplemental material. Download SPECTRUM00571-21_Supp_1_seq25.pdf, PDF file, 1.1 MB [file spectrum00571-21_supp_1_seq25.pdf]

Fig. S1

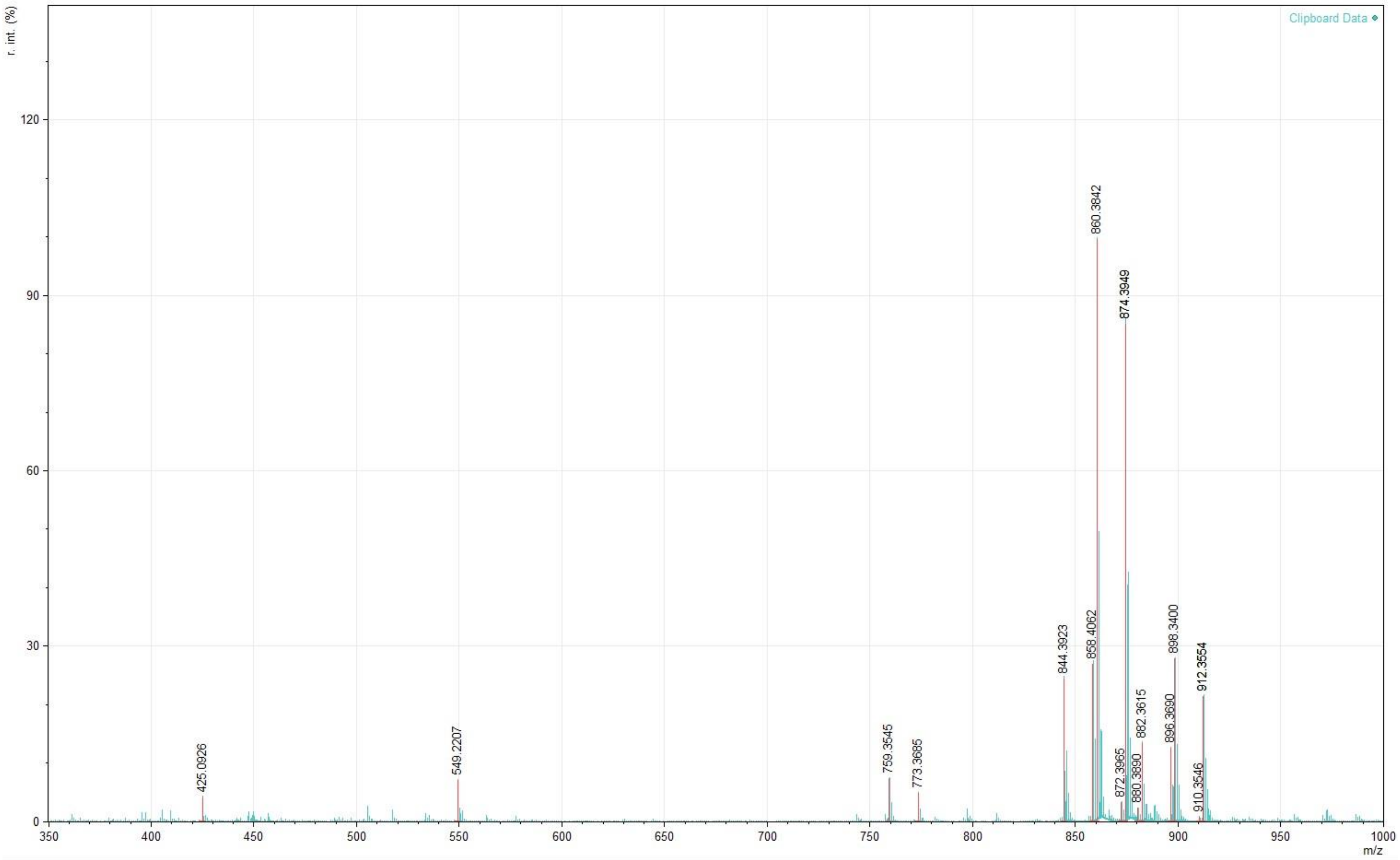

Figure S1: Full-scan MS spectrum of the 5.52 min elution fraction. Rotihibin C: m/z 860.38 $[M+H]^+$, m/z 882.36 $[M+Na]^+$, m/z 898.34 $[M+K]^+$; Unsaturated rotihibin C: m/z 858.41 $[M+H]^+$, m/z 880.39 $[M+Na]^+$, m/z 896.37 $[M+K]^+$; Rotihibin D: m/z 874.39 $[M+H]^+$, m/z 896.37 $[M+Na]^+$, m/z 912.36 $[M+K]^+$; Unsaturated rotihibin D: m/z 872.40 $[M+H]^+$, m/z 910.35 $[M+K]^+$; Rotihibin variant with shorter acyl chain: m/z 844.39 $[M+H]^+$, m/z 882.36 $[M+K]^+$.

Fig. S2

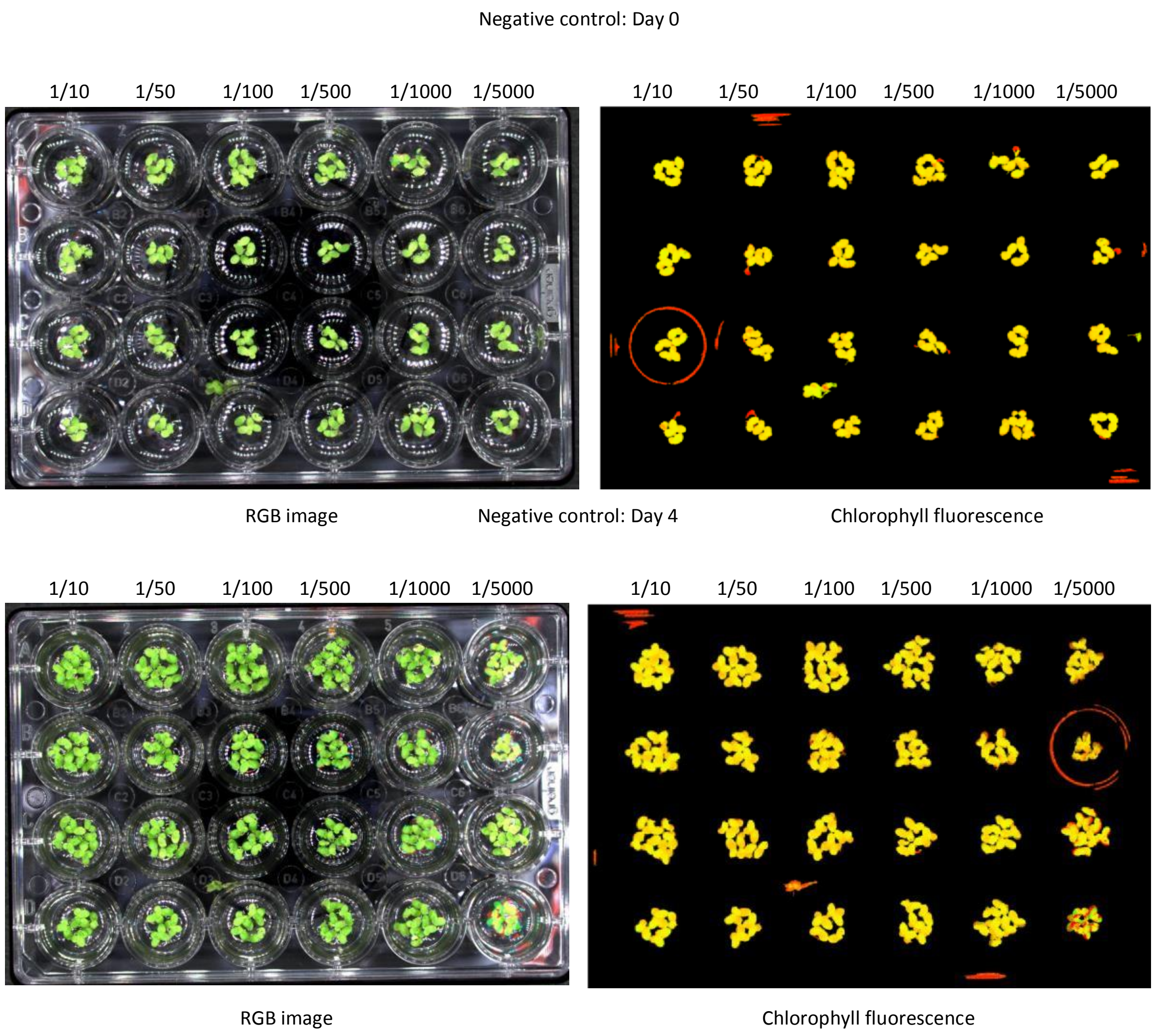

Figure S2: *Lemna minor* bioassay results, negative control (neighbouring HPLC peak)

Fig. S3

Rotihibin C (µM): Day 0

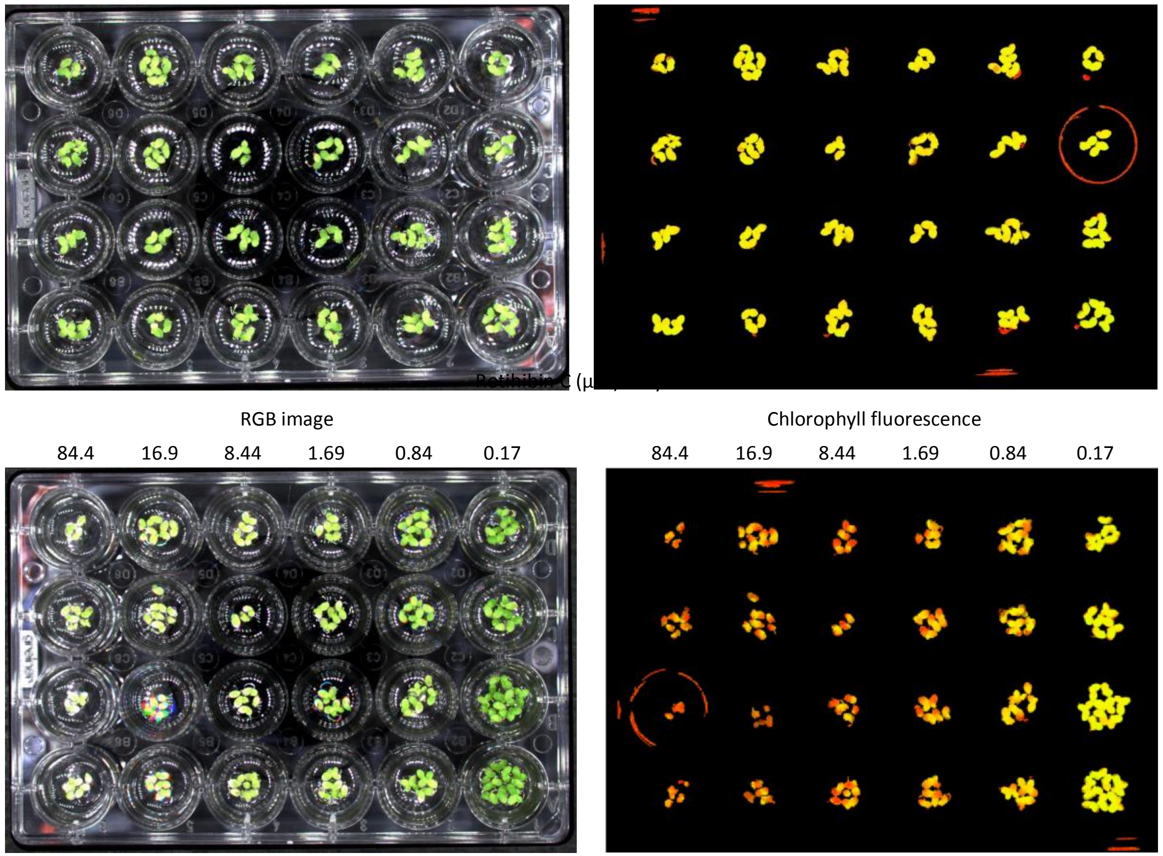

Figure S3: *Lemna minor* bioassay results, rotihibin C

Rotihibin D (µM): Day 0

Fig. S4

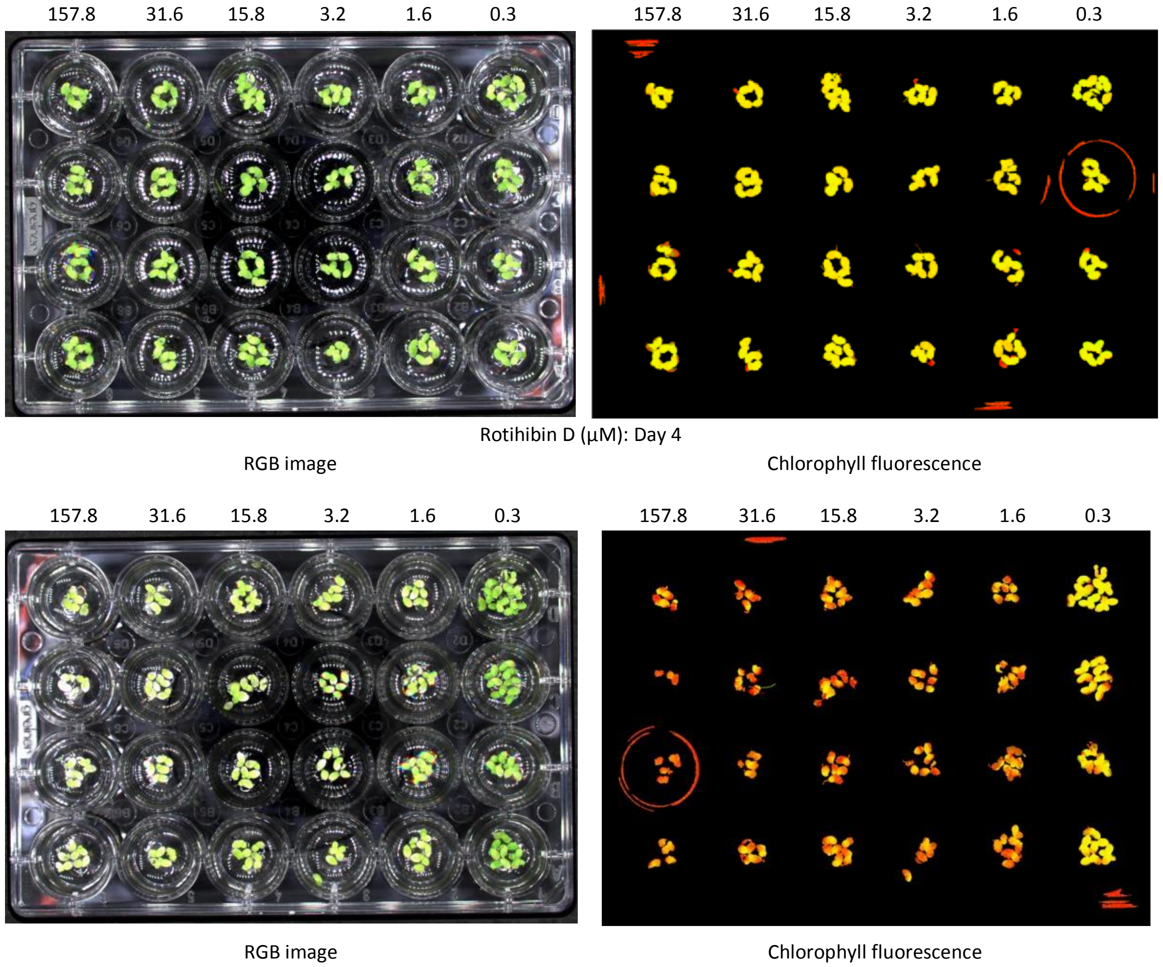

Figure S4: *Lemna minor* bioassay results, rotihibin D

Fig. S5

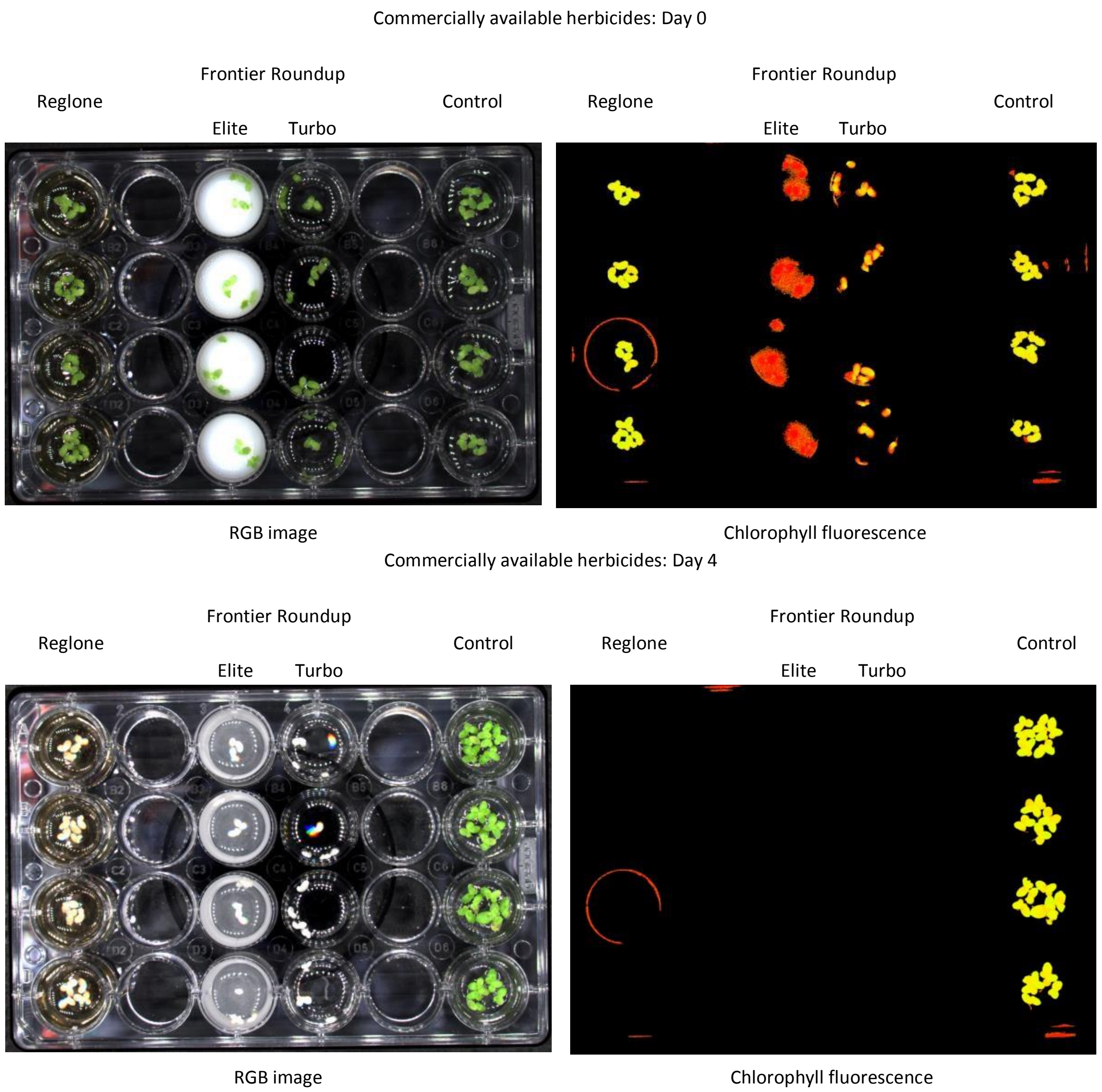

Figure S5: *Lemna minor* bioassay results, commercially available herbicides
